# Fast and effective assessment for individuals with special needs form optimization and prediction models

**DOI:** 10.1186/s40359-025-02768-z

**Published:** 2025-04-22

**Authors:** Bilal Baris Alkan, Muhammet Kumartas, Serafettin Kuzucuk, Nesrin Alkan

**Affiliations:** 1 Measurement and Evaluation in Education, Faculty of Education, University of Akdeniz, Dumlupınar Boulevard, Campus, Antalya, 07058 Turkey; 2 Guidance and Psychological Counselling, Beyşehir Guidance and Research Center, Konya, 42700 Turkey; 3 Faculty of Economics and Administrative Sciences, Akdeniz University, Dumlupınar Boulevard, Campus, Antalya, 07058 Turkey

**Keywords:** Assessment, Special needs, Prediction, Model, Machine learning

## Abstract

The aim of this study was to determine which items in the psychological assessment forms used by counselling and research centres for individuals with special needs are effective in classifying individuals into special needs diagnostic categories. Data were obtained from the psychological assessment request forms of 1814 individuals aged 6 to 12 years who were referred to the centre between 2019 and 2023 with suspected special needs and who were classified as having special needs based on psychological and developmental assessments. In addition, we wanted to develop new predictive models using machine learning methods based on these items. Optimizing the psychological assessment application form so that it contains fewer questions may help to make the assessment process faster and more effective. It is expected that the results of this study will make an important contribution to saving time and energy for experts and individuals.

## Introduction

Today, approximately 13% of people between the ages of 6–18 in modern education systems are individuals with special needs [1–4]. Assessment of individuals with special needs is critical in several areas, including cognitive skills, daily living skills, speech and language development, and social interaction. Standardised tests are widely recognised as basic tools for assessing these areas. However, recent studies suggest that the broad range of skills required cannot be effectively assessed by a single measurement tool or professional [5, 6]. These findings emphasize the importance of using multidimensional data and an interdisciplinary approach to psychological and educational assessment processes.

Psychological diagnostic tests provide valuable insight into individuals; however, misapplication can lead to poor decisions that can have a detrimental impact on the lives of those affected [7]. A well-structured psychological assessment process ensures accurate identification of the individual’s current level of performance and needs, facilitates the planning of appropriate educational programmes and leads to productive outcomes for both the individual and their community. Conversely, inaccurate assessments can result in the denial of appropriate educational opportunities, social environments and necessary services [8]. Consequently, a multifaceted approach to assessment processes is essential.

Effective assessment requires the consideration of data from different social environments and multiple sources of information about the individual at each stage of the assessment process. However, methods that go beyond standardised tests and pre-assessment data are often not adequately integrated into these processes [9, 10]. The administration of standardised tests is time-consuming; the total time required for diagnostic procedures per person is between 4 and 14 h, with an average of 11 different tests or inventories used. The limited availability of assessment professionals further exacerbates this workload, resulting in underutilisation of pre-assessment data. In addition, challenges such as inappropriate measurement and assessment environments, reliance on outdated instruments, lack of collaboration and poor adherence to testing protocols when using standardised tests can lead to inaccurate results [11]. These problems highlight the significant limitations of current psychological and educational assessment methods, particularly the underutilisation of pre-assessment data.

The aim of this study was to determine which items in the psychological assessment forms used by the counselling and research centres for people with special needs are suitable for assigning people to the diagnostic classes for special needs. In addition, new prediction models based on these items should be developed using machine learning methods. Optimising the application form for psychological assessment so that it contains fewer items can help to make the assessment process faster and more effective. It is expected that the results of this study will make an important contribution to saving time and energy for experts and individuals.

In the rest of the paper, the data source, data processing steps, and application details of the machine learning models will first be presented in detail. Then, the results of the analysis will be discussed based on the performance metrics obtained during the model validation process, and the best-performing method will be highlighted. In the final section, the contribution of the findings to practical applications, the study’s limitations, and suggestions for future research will be evaluated.

## Theoretical background

### Individuals with special needs

Individuals with special needs differ from their peers in physical, mental, social and emotional areas that can be assessed using measurement tools depending on various factors that influence the developmental process. These individuals require specialised support in areas such as education, health care and social services. To assess individuals with special needs, psychological and educational assessments are carried out by psychological counsellors, psychologists and guidance teachers using standardised measurement tools. Based on the data obtained, the individual’s psychological and educational needs are identified and plans are developed that include the necessary adaptations to fulfil those needs. The objective, comprehensive, consistent, and functional implementation of this critical process directly impacts all aspects of an individual’s experience and quality of life [12–15].

Today, many educational systems continue their studies to define specific requirements and determine standards for the psychological assessment of individuals with special needs. However, the assessment procedures for the clinical and educational diagnosis of individuals with special needs are still not clear. There is an increasing need for different perspectives on the psychological assessment of these individuals, and new diagnostic and assessment methods that can be applied in educational systems [16–19].

The classification and terminology used in the psychological diagnosis of individuals with special needs may vary from country to country and region to region. These differences are influenced by cultural, legal and educational policy conditions. In industrialised countries, specific learning disabilities, intellectual disabilities and autism spectrum disorders are common diagnostic categories. Attention-deficit/hyperactivity disorder (ADHD), emotional or behavioural disorders and speech and language disorders are also frequently diagnosed. In addition, physical disabilities, sensory impairments (e.g. visual or hearing impairments), developmental delays and other health-related disorders are also recognised as part of special needs [20–24]. While definitions of each special needs category are provided in the clinical classification procedures, there is often no general guidance on which tests or procedures are used to determine classification in each clinical diagnostic category. These decisions may vary from country to country, from region to region and from stage to stage of development. New methodological approaches that can be used in virtually all educational systems, are easily adaptable to all developmental levels and different cultures, and have high classification accuracy are increasingly being investigated [21, 22, 24].

## Psychological and educational evaluation process

The process of assessing individuals with special needs in many educational systems requires co-operation between professionals and institutions. The specific centres or professionals involved in the assessment may vary depending on the nature of the individual’s needs and the resources available in the community. However, in any country with a modern education system, there are a number of diagnostic centres, clinics and research facilities that provide guidance and counselling services in the fields of education, psychology and special education. These centres include professionals such as psychological counsellors, guidance teachers, psychologists, speech pathologists and occupational therapists who work together to assess the needs of the individual [25–28].

Initially, those affected were referred to these centres for clinical and educational assessments. A parent, teacher or other professional may refer a person with special needs to these centres for a psychoeducational assessment. The referrals usually contain a large amount of unstructured information about the individual, primarily gathered from parents and teachers, which is used in the subsequent assessment process. This information consists of various checklists, portfolios and observation forms [29]. In these centres, the evaluation process is then completed using standard tests and diagnostic methods to conduct a psychological assessment of the individuals and make pedagogical decisions. Educational and psychological decisions are then made according to the standards set for the individuals concerned. However, the extent to which the established standards are implemented varies from country to country [14, 30].

To ensure that important psychological and educational assessment decisions for people with special needs are valid, it is important to involve a group of more than one expert and to incorporate information from different psychosocial environments into the assessment, rather than having a single expert conduct the psychological assessment and use a limited measurement environment. However, a lack of procedural fidelity and systematic decision making has been observed in such multifaceted assessment methods [31]. In some cases, the failure of experts to conduct psychological assessments leads to significant problems. Previous studies have found that team-based decisions lead to limitations when looking at data from a holistic perspective, potentially reducing classification accuracy and validity [28]. New methods for objectively evaluating the accuracy of psychological and educational assessments and psychological data from different sources can be proposed.

## Methods used in psychological and educational evaluation

Numerous studies have investigated the technical appropriateness and suitability of methods for the psychological and educational assessment of individuals with special needs. Most of these studies have focussed on the theoretical perspectives in the psychological assessment of individuals with special needs. The norm-referenced approach, the continuous observation approach, and variations of these two basic approaches are supported by various researchers on the assessment of individuals with special needs [10, 13, 32–36]. In all of these approaches, the use of standardised tests and assessment methods is seen as a primary [33–35]. In various areas of special needs such as intellectual disability, specific learning disability, physical disability and autism spectrum disorder, many standardised tests such as WISC-R, Stanford-Binet, Kaufman Assessment Battery for Children (K-ABC) and Bender Visual-Motor Gestalt Test are used [37–40].

It is generally recognised that such tests are the most important source of data for psychological assessments. However, there are several problems associated with the effective use of such measurement tools. Firstly, the suitability of the measurement instruments, the appropriateness of the physical conditions in the measurement environment and the standardisation of the language in which these instruments are used are prerequisites for a healthy assessment [41, 42]. However, in some studies, inadequate and outdated measurement instruments were used and the physical conditions of the test environment were not suitable [43–45]. In addition, testing procedures vary according to the different demographic characteristics of the individual, which complicates the work of the experts conducting the psychological assessment. All these problems can lead to errors in the psychological assessment processes of people with special needs.These problems are listed as follows:

- *General limitations of measurement and assessment tools*: The tests and assessment tools used are outdated, cannot be adequately adapted for different cultural and linguistic groups, and are not suitable for the diversity of individuals with special needs.
- *Lack of measurement tools suitable for the specific needs of the assessment units (i.e. the specialised departments within the counselling and research centres that carry out psychological assessments)*: There is a lack of valid and reliable measurement instruments specifically designed and optimised for the particular needs of each assessment unit, taking into account factors such as the target population, available resources and testing conditions.
- *Technical and physical inadequacies in assessment environments*: Inadequate supply of equipment, sub-optimal physical conditions and lack of technological infrastructure in assessment processes.
- *Inadequacy of expert staff and lack of training*: The small number of experts working with individuals with special needs, the lack of adequate training in measurement and evaluation processes and limited methodological knowledge on test applications. In addition, inadequate teamwork in assessment processes and weak interdisciplinary co-operation processes.
- *Methodological inconsistencies in implementation processes*: Inconsistencies in evaluation methods and protocols between different units, which adversely affect the reliability and comparability of evaluation results.
- *Insufficient adoption of ethical principles in test applications*: Inadequate application of ethical standards in testing processes, failure to observe the rights of individuals and the emergence of problems related to data security.
- *Insufficient time allocated to individuals in educational assessment processes*: Individuals are not given enough time in order to speed up the assessment processes, which negatively affects the accuracy and reliability of the measurement.
- *Problems in the application of psychometric criteria to test scores*: Inadequacies in the validity and reliability analyses of measurement tools, the use of non-standard approaches in the interpretation of scores and the lack of scientific support for the evaluation results.
- *Inconsistencies in the integration of information obtained from different data sources*: In the evaluation processes, data collected from more than one source cannot be evaluated in integrity, data integration deficiencies and contradictory results emerge.
- *Digital applications and technological infrastructure deficiencies*: Insufficient integration of digital systems in evaluation processes, lack of technological support mechanisms and ineffective use of digital tools.
- *Financial resource constraints*: Failure to ensure cost-effectiveness in the procurement, implementation and analysis processes of measurement tools, and inability to carry out evaluation processes at an ideal level due to economic constraints.

These problems have been highlighted in various scientific studies [6, 19, 41, 42, 46–53]. Therefore, relying heavily on test scores for educational and psychological assessments of these individuals can be problematic. In practise, these drawbacks severely limit the psychometric quality of the tests. Eliminating all these limitations requires considerable time and financial resources and necessitates the search for new measures and approaches that are alternatives to standard tests and procedures.

## Leveraging Pre-Evaluation data

When making decisions on important issues such as psychological diagnosis and educational placement, collecting information about various developmental areas from the individual’s social environment, observing the individual’s behavior in different social environments, and extending the measurement and evaluation process over a certain period of time is extremely important to conduct the evaluation appropriately [54–56]. It is very important to understand the importance of stakeholders who make up the individual’s social environment, especially parents and teachers, as data providers in psychological and educational evaluations. Families and the educational environment generally have more information about individuals than experts who conduct clinical evaluations. This valuable information can be very helpful in the psychological evaluation of individuals with special needs [57, 58]. In their research [59], revealed the factors that affect the school selection of individuals with special needs and determined that one of the seven most effective factors among the 2,662 articles they examined should be the information provided by the family and educational environment about the individual before psychological and educational diagnosis.

However, several studies suggest that pre-assessment data, which is a very important data source for the psychological and educational assessment of individuals with special needs, is not being utilized well enough. A recent study reported that school counselors across the United States reported that test scores were the most important data source for the educational assessment of individuals with special needs, followed by progress monitoring, record reviews, and developmental history. Very few participants (6%) reported that pre-assessment data were equally important [10, 60]. found that most individuals referred to the grounds that they had special needs (92%) were subjected to a standard test at the relevant centers, the vast majority of those tested (73%) received a psychological diagnosis based solely on test scores, and other criteria were disregarded in the evaluation. In studies conducted with different groups in Turkey, it was determined that experts working in Guidance and Research Centers could not allocate the necessary time for measurement and evaluation processes because of the heavy workload due to insufficient personnel. Although various preliminary information were collected from the family and school, it was determined that most of the time, they made educational evaluation decisions based on the results of a single standard measurement tool without using these data [11, 61–64]. Other studies have shown that, despite the rationale and comprehensive data written in the guidance forms before educational evaluation, this information is not sufficiently utilized before psychological diagnosis [9, 65, 66]. Various machine-learning algorithms may be utilized to make such input data useful for the psychological evaluation of individuals with special needs.

## Supervised classification

The first step in supervised classification methods is to determine the classification problem. Defining the classification purpose, determining the objectives and evaluating the classification results constitute the next steps. After the classification problem is determined, the data preprocessing process starts [67]. Here, the missing data problem is solved first. Then, it should be checked whether the data set contains balanced samples according to the label classes. One method to reduce or eliminate class imbalance is SMOTE. This technique was applied to the six-class, high-volume datasets used in our study. The next step was data normalization, also known as feature scaling, which involves transforming all input data to a common scale. The variables used for classification were then selected from the independent input variables to improve inference from the classification model. Several methods have been proposed for this purpose [68].

In the study, not all input data were included in the model; only those that significantly contributed to the classification were selected.

After these processes, the classification solution has two remaining steps: a training step and a test step. In the training step, the classification model is created. In this step, it can also be defined as finding a mapping or function that can predict the class label of the input data. In this step, a relatively large part of the available dataset (the training dataset) was used. In the test step, the established model predicts the class labels of a specific dataset. In the second step, the prediction accuracy of the classifier was investigated. In this stage, the dataset that was not used in the first step (test dataset) was used to avoid overlearning. The classification accuracy was then examined against various outcome measures. The accuracy of the classifier is the percentage of test data correctly classified by the model. If the accuracy of the classifier is considered acceptable, the resulting model can be used on future datasets where the class labels are unknown [69]. For individuals with special needs in our study, the data obtained before the psychological and educational evaluation (data in referral and orientation forms) constituted the input education data to be included in the classification, and the diagnostic categories determined by the relevant centers and experts as a result of clinical and educational diagnosis constituted the class labels.

For the supervised classification methods, the predicted classes were based on well-trained models. In our study, the psychological and educational assessments of individuals with special needs were tested using more than one model that provided strong classification accuracy in the literature. Simultaneously, a similar approach was adopted for the parameters determined for the models and training strategies. The estimation models used in the study, selected parameters, and algorithm stopping rules are given in the following sections. Some of the concepts discussed in the relevant section are briefly discussed below.

### Data normalization

To prepare the data for classification, bringing all data to the same range is a very important step in the data preprocessing process. Scaling methods such as scaling by unit length, scaling by variance, scaling based on mean, TanH, scaling by maximum and minimum values, ​​and T-score have attracted much attention in practical use [70, 71]. In this study, the input data were scaled according to the minimum and maximum values. This means that all independent variables were scaled to the range (0, 1) with (x-min(x))/[max(x)-min(x)] in the calculation. This type of scaling provides easily comparable results. In addition, the fact that it can scale categorical item responses and is easy to calculate makes it suitable for our research data in practical terms.

## Feature selection

Feature selection is an approach that can be efficient in terms of creating a less complex model, making correct inferences from the model, and computational cost. In this study, a Recursive Feature Elimination method was used for the feature selection. Recursive feature elimination is an effective method widely used in feature selection. The main purpose of Recursive Feature Elimination is to determine the importance coefficients of independent variables according to class labels, and to determine the variables that contribute the most to the model, starting from the most important predictor variables. The selection of variables is done recursively, the reason for this is that the importance of some variables may vary according to different subsets [72]. Recursive Feature Elimination allows us to determine the optimum subset that best represents the input dataset by gradually eliminating features that contribute the least to the model [73]. As is well known, filter methods such as correlation analysis rank independent variables according to their individual relationships with the target variable, but ignore interactions that occur during model training. This may limit the capacity to capture multiple interactions in the high-dimensional and multi-class psychological pre-assessment form used in our study. Similarly, wrapper techniques such as embedded methods or forward/backward selection offer approaches that are difficult to integrate into model performance and require high computational cost, making practical applicability difficult and limiting the interpretability of results in our high dimensional data set of 171 items. For this reason, the systematic approach of RFE in determining the variables that directly contribute to the overall performance of the model has been decisive in its preference.

Furthermore, since the Repeated Feature Elimination (RFE) method is integrated with k-fold cross-validation in this study, the performance metrics of the model (e.g. accuracy, Kappa, cross entropy) are carefully monitored at each iteration. In this way, the impact of removing poorly correlated features on model performance is continuously evaluated. That is, at each step in the RFE process, the change in the overall predictive accuracy of the model is observed; if the removal of a particular feature group leads to a significant decrease in model performance, this indicates that those features may be important. This approach provides an evaluation mechanism similar to sensitivity analysis to avoid inadvertent elimination of features that have low correlation but contribute to the generalisability of the model [74, 75].

## K-Fold cross validation

A k-fold cross-validation validation method was used to minimize sampling bias. To minimize sampling bias, this method first divides the data into k-sized pieces. It separates one of these pieces for testing and uses the remaining k-1 pieces for training. Its advantage is that all observations were used for both training and validation, and each observation (k piece) was used once for validation. In the k-fold validation model, the k value is widely selected in the literature as 5 or 10, which is considered to be the ideal rate for processing complexity and validation [76, 77].

### Performance metrics

Various metrics have been used to examine the accuracy of the classification algorithms. In our study, Accuracy, Kappa and Cross-Entropy metrics, which are widely used as classification accuracy measures, were considered.

#### Accuracy

It measures the proportion of examples correctly predicted by the classification model. This was expressed as a percentage. It is calculated using the formula Accuracy = number of correct predictions/total number of predictions. Accuracy is a widely used metric for evaluating the overall performance of a model [78].

#### Kappa

Cohen’s Kappa coefficient is a statistical method that measures the reliability of agreement between two raters. Similar to most correlation statistics, Kappa can range from − 1 to + 1. Kappa = 1 indicated perfect agreement, Kappa = 0 indicated random agreement, and Kappa = -1 indicated complete disagreement. Generally, a Kappa value greater than 0 indicates agreement between raters. However, it is recommended that agreement be taken into account when the Kappa value is 0.80 and above [79].

#### Cross-entropy

It is a probability-based function used to evaluate the model performance in classification problems. The probability function was calculated using the model outputs and class labels, and the model parameters were updated to minimize this value. In a multiclass dataset, p(x) represents the true distribution of the data and q(x) represents the class distributions predicted by the model. The cross-entropy value between these distributions was calculated using the formula: H(p, q)=−∑p(x)log(q(x)). If p(x) = q(x), the cross-entropy value is zero, which indicates that the predictions completely match the true distribution. However, if p(x) and q(x) differ, the cross-entropy value is positive. In other words, more accurate predictions resulted in lower cross-entropy values ​​ [80, 81].

#### Sensitivity

*This refers to the ability to correctly identify true-positive results in classification problems.* Mathematically, sensitivity is calculated by dividing the number of true positives by the sum of the numbers of true positives and false negatives and takes a value between 0 and 1. *Higher values ​​indicate a better classification accuracy.*

### Supervised classification algorithms

Supervised classification methods have been used for the clinical diagnosis and assessment of people with special needs. These methods are frequently used in the psychology and education literature. They are also considered robust classification methods, which makes them suitable for the purposes of this research. The choice of K-Nearest Neighbour (KNN), Random Forest (RF), Naïve Bayes (NB) and Support Vector Machines (SVM) models in this study is based on practical factors such as structural features of our dataset, ease of implementation and reproducibility. KNN provides fast classification of categorical data, especially in psychological evaluation forms, thanks to its non-parametric structure and working with basic distance metrics. This method, which makes it easier for experts to understand and discuss classification decisions, can also be easily implemented in the R programme with extensive package support [82]. The RF algorithm offers guidance to researchers seeking to optimise form, by automatically highlighting critical variables in determining diagnostic classes [83]. Furthermore, although not directly necessary for our current dataset with no missing data, the robustness of RF to missing data may provide flexibility for future work. NB is characterised by its high performance even on small data sets and its low computational cost, making it applicable in resource-limited environments [84]. Thanks to its natural fit with categorical responses, which are frequently encountered in psychological measurement tools, it offers a model that is easy to interpret for experts with limited technical knowledge. SVM is notable for its high classification success in complex data structures and tools that allow hyperparameter optimisation. It has a strong discrimination capacity, especially between categories that are difficult to distinguish, such as autism spectrum and intellectual disability. These selected models have strong theoretical underpinnings and empirical findings in the machine learning literature [85, 86] strengthening the methodological consistency and applicability of our study. The methods are briefly described in this section.

#### KNN classifier

The K-Nearest Neighbor classifier is one of the most effective and efficient supervised classification algorithms that are classified based on distance and are frequently used in classification and regression problems [87, 88]. KNN generates a prediction function from a training dataset containing input and output variables, which are then classified and assigned to a group according to this prediction function. To determine the nearest neighbor, a distance measurement is made between the unlabeled observation and the examples in the training set, and a new example is assigned to the category that provides the majority of the nearest neighbors. In other words, instead of considering a single example closest to a unit in its classification, it is based on multiple neighbors. K is the number of nearest neighbors of the unlabeled observations in the majority. To determine the K value, the values ​​are tested in order, starting with K = 1, and this continues until the K value that gives the lowest error value in classification accuracy is reached. K is usually a single number, and the larger the training dataset, the larger is the K value. Distance measures were used to include unlabeled observations in the classification prediction function. These measures are distance measures, such as Euclidean, Hamming, Manhattan, and Minkowski. The KNN algorithm focuses on the similarity between unlabeled observations and classified observations and assigns a new observation to the class with the observations that it is most similar to [89].

#### Random forest classifier

The Random Forests classification algorithm is an ensemble learning method that was developed by [90]. It is based on a holistic view of the bagging method developed by [91] and The Random Subspace method proposed by [92]. The difference from the bagging method is that it creates subgroups in variable selection. The trees created were obtained with bootstrap samples at each node and n estimators, which is less than the total number of estimators (N), and no pruning was applied to the created decision tree (N > n) [90]. In this classification method, tree-type partitioned classifiers formulated as {h(x,θk) k = 1,…} were used. Here, x represents the input data, θk, and represents the random vector. Different datasets smaller than the basic dataset are created, and new trees are grown in these subsets by the random feature selection method. The number of trees and variables to be used in each node are determined by the user, and then the number of variables is increased or decreased until the errors are reduced to the optimum level. The number of variables that are less or more than the optimum value has a linear effect on power and correlation. The algorithm divides the predictors in a binary manner and only two subnodes are formed from each parent node. The Gini index is used in this division process; it is kept at a minimum value to ensure class homogeneity, and the classification is completed when it reaches zero [93, 94].

#### Naive Bayes classifier

Bayes’ theorem, based on the probability theory, shows the relationship between conditional probabilities and marginal probabilities for any random variable in a probability distribution. It is an estimator and classifier algorithm that analyzes the relationship between a target variable and independent variables. The probability value of a conditional event A is different from the probability value for a related event B. In other words, the probability of event B occurring when event A occurs and the probability of event A occurring when event B occurs are different, but there is a connection between these two conditional events. It is an algorithm that relates the conditional probabilities of two random events based on the maximum likelihood principle and estimates examples of the class with the highest probability [95–97]. There is widespread consensus that it is more functional than other algorithms owing to its ease of application and its useful and powerful nature in big data. Another advantage is that it can produce high classification accuracy results with small training data without the need for very large datasets, and can be used with continuous or discrete [98, 99].

#### Support vector classifier

Support Vector Machines are powerful machine learning algorithms used in classification and regression problems. Support Vector Classifiers (SVC) have been developed to classify multiclass and nonlinear data. The basic principle is to divide the data points with a hyperplane that provides the best separation between the classes. This hyperplane attempts to maximize the margin between classes and is also based on data points called support vectors [100]. One of the main features of the SVC is that it can handle nonlinear classification problems using the kernel method. The kernel method increases linear separability by transforming data points into a high-dimensional feature space. In this manner, it can also work effectively on datasets with complex structures. This classification algorithm allows very successful results to be obtained on the generalizations of multilayer input transformations [101].

Its advantages include effective generalization ability, the ability to work with high-dimensional datasets, and the ability to be effective in high-dimensional feature spaces, even with low-dimensional examples. However, the training time of SVC can be high for large datasets, and tuning the hyperparameters of the model can sometimes be challenging. However, when configured correctly, SVM can deliver excellent results in many complex classification and regression problems [102, 103].

One of the most important issues that researchers working in the field of psychological and educational assessment focus on is the research conducted on the effectiveness and functionality of methods related to the diagnosis and classification of individuals with special needs [12, 104]. There are many different methods based on theoretical and formal methodologies for the educational diagnosis of individuals with special needs. Each method has certain advantages and disadvantages, including basic assumptions and various standardized criteria. However, there is no consensus on the best method for addressing this issue.

This study proposes the use of various machine learning methods for this purpose, and aims to include all complementary pieces of information obtained during the psychological assessment process of the individual. A psychological and educational diagnostic method based on machine learning can provide practical solutions by saving time and resource-intensive assessment processes.

The diagnostic areas for individuals with special needs cover a wide range. The psychological evaluation of these individuals requires the use of different measurement tools for each diagnostic class and the evaluation to be carried out by different experts.

When it comes to the clinical assessment of individuals with special needs, this is not synonymous with testing. Instead, it refers to the process of collecting data to make decisions regarding individuals. This process accounts for a large portion of formal pre-assessment data that is not sufficiently used for psychological diagnosis. The general aim of our study was to create assessment models with high predictive accuracy, where pre-assessment data were used effectively. In addition, we suggest a procedure for structuring pre-assessment forms for each educational system, region, and culture. An alternative method with high predictive accuracy, including useful and versatile information and well-explained application procedures, could be an indispensable tool for researchers responsible for the psychological and educational assessment of individuals with special needs.

### Methods

#### Data source

The data used in our study were obtained from a Guidance and Research Center responsible for a comprehensive region in Türkiye. These centers are mostly responsible for various counseling services in the fields of psychology, education, and the psychological diagnosis of individuals with special needs. In many educational systems, there are Guidance and Research Centers or different centers or health institutions that perform the same functions under other names. The data were obtained from the psychological evaluation request forms of 1814 individuals between the ages of 6–12 who were referred to the center with suspicion of having special needs between 2019 and 2023 and who were classified as having special needs as a result of psychological and developmental evaluations. This form provides basic information to identify the difficulties experienced by individuals and directs them to appropriate support services. These forms are based on observations of individuals obtained from multiple sources of information in different social environments over a period of at least six months. The forms in our study included observation lists filled out jointly by teachers, parents, and school counselors. These lists include preliminary diagnostic data with 171 items answered categorically (yes, sometimes, no) in many developmental areas, such as cognitive abilities, visual perception, attention, auditory perception, language and speech, early literacy, psychomotor skills, problem solving, self-care, and social skills.

In addition, this study included diagnostic classes, which are psychological and educational evaluation decisions made about individuals as a result of clinical and educational evaluations conducted in the centers. Special needs evaluations were conducted for six classes in these centers. These classes included six different categories in the areas of specific learning difficulties, mental disabilities, physical disabilities, autism spectrum disorders, visual or hearing disabilities, and behavioral and adaptation problems. Therefore, it is important to note that there were six different class labels in our study. These class labels were used to determine the educational and psychological needs of individuals and create a framework for the provision of appropriate services. Each class label is used to define the special needs of individuals in a certain category, which helps educational and psychological support providers determine appropriate interventions. The special-needs class labels are listed in Table 1.

**Table 1:** Special needs class labels

Special Needs Class Labels	*N*
Mental Disability	373
Behavior and Adaptation Problems	350
Autism Spectrum Disorders	324
Physical Inadequacy	183
Visual or Hearing Impairment	176
Specific Learning Disability	408
Total	1814

#### Data processing

There are no missing values in the study data. In order to assess the balance of the data set, the distribution of classes was analysed in detail. In this direction, imbalance ratio, entropy, Gini coefficient and Chi-Square analyses were performed. The calculated imbalance ratio value was found to be 2.318, which indicates the presence of a moderate imbalance between classes as stated in [105]. The entropy value was calculated as 1.7437 and it was concluded that the distribution of classes in the data set showed diversity according to [106] principles. The Gini coefficient is 0.1613, indicating that the distribution of classes is moderately homogeneous, as interpreted by [107]. As a result of the Chi-Square test (χ² = 162.41, df = 5, *p* < 0.001), a significant difference was found. This result, on the basis of [108], shows that there is a statistically significant distribution difference between the classes and therefore the data should be balanced.

SMOTE (Synthetic Minority Over-sampling Technique) method was used to eliminate the existing imbalance in the data set and to balance the sample distribution between classes [109]. The SMOTE algorithm generates new synthetic examples by considering the nearest neighbours of the examples in the minority classes in the feature space. This technique is an effective method for reducing imbalances in the data set and helps to minimise biases in the subsequent model training stages. R programming language and smotefamily package were preferred in the analysis process [110]. The number of nearest neighbours (K) was set as 5 as a hyperparameter, and the number of samples of each class in minority classes was increased to 408 samples to equal the majority class. In order to ensure reproducibility during the analysis, the random state value was fixed as 42. As a result of this process, a new balanced data set consisting of 2448 samples, which is a combination of synthetic and original samples, has paved the way for more reliable and unbiased results in subsequent analyses.

Once the data were scaled and normalised to minimum and maximum values, the process of feature selection was initiated. One of the specific objectives of our research is to present various procedures for the effective use of forms based on similar prior knowledge in different educational systems and regions in the context of psychological assessment. Accordingly, the aim of feature selection is to achieve maximum classification accuracy with an optimum number of variables. For this purpose, the number of cross-validations was used as the first criterion in the iterative feature selection process. As an external resampling method, the results of multiple parameters were examined in the ‘caret’ package of the R programme [111], starting from double-fold to 10-fold cross-validation; other parameters were kept constant.

The number of repetitions for each trial was set to five and the training percentage for out-group cross-validation was set to 0.80. In the implementation of feature elimination, the random forest function (rfeFuncs) was used for training and validation of the model, and the accuracy and Kappa metrics were chosen as the basic parameters to determine the model accuracy. Furthermore, the consistency of the interpretation was improved by using a random forest classifier based on the default parameters for each k-fold cross-validation value and cross-entropy metric. In the final stage, 80% of the data according to the label classes were used for training the supervised classification algorithms, while the remaining 20% was used for model validation and determination of the classification detection rate.

The preference for the min-max scaler in the data preprocessing stage was decided by considering the methodological consistency of the study and the specificity of the data structure. Firstly, normalising features to the interval [0, 1] prevents high-scale variables from dominating model decisions, especially when distance-based algorithms (e.g. K-Nearest Neighbour) are used. This approach supports balanced learning of the model by allowing all features to contribute equally. Secondly, unlike standard scaling, the min-max method has the advantage of preserving the shape of the original distribution of the data. Since the psychological assessment data used in the study have natural boundaries such as Likert-type scales and categorical responses, not distorting the distribution is critical for data integrity [86]. Thirdly, the simple mathematical formulation of min-max scaling (x - min(x))/(max(x) - min(x)) provides resource efficiency by reducing computational complexity in large data sets [85]. For these reasons, the min-max scaler was preferred due to its both theoretical and practical suitability.

The aim of feature selection is to achieve maximum classification accuracy with the optimum variable set, especially in the context of creating an effective preliminary psychological assessment tool presented in this study. For this purpose, the RFE method was used. RFE helps to determine the optimal feature subset by iteratively eliminating the variables with the lowest contribution according to the variable importance levels of the model. In order to perform feature selection in the best way, model accuracy rate and AUC values ​​were taken into account in the RFE process and the most appropriate parameters were determined. Model training was initially performed with 171 features, then the number of features was gradually reduced and different subsets were evaluated. The elimination process was carried out gradually and balancedly by applying a 5% feature elimination rate in each iteration. When deciding which variables to keep, the “Mean Decrease in Gini” (MDG) metric was used. In order to verify the generalization performance of the model trained with the selected feature set, multiple parameter combinations were examined with 10-fold cross-validation starting from double-fold in the R program ‘caret’ package [111], the number of repetitions for each trial was fixed as 5 and the training percentage as 0.80.

All these methodological steps ensured that the dataset would form a solid foundation for the models to be created in the following stages and contributed to the model achieving high classification accuracy while preserving the structural integrity of the data. The obtained balanced dataset and the optimum feature subset support the model to provide more reliable and unbiased results in both academic studies and practical applications.

In summary, the absence of missing values ​​in the study, detailed analysis of the distribution of classes with various statistical methods, elimination of imbalances with the SMOTE method, normalization of the data with the min-max scaling method and determination of the optimal feature subset with the RFE method allowed the model to achieve high classification accuracy. However, the fact that the dataset is limited to a specific geographic region and children between the ages of 6 and 12 necessitates additional research on whether the model will perform similarly across different populations. In addition, issues such as demographic fairness, minimizing expert biases and overfitting risks during the integration of the model into real-world applications are among the important issues that further studies should address. Therefore, it is expected that future studies will contribute to increasing the generalizability of the model by testing it on multi-center datasets with different demographic characteristics and to developing methods that will produce more comprehensive and reliable results in psychological and educational assessment processes.

### Classification

After recursive feature elimination, the R Program ‘class’ package [99] was used for the KNN Classifier, the R Program ‘randomForest’ package [100] for the Random Forest Classifier, and the R Program ‘e1071’ package for the Naive Bayes and Support Vector Classifiers [101]. Hyperparameter selection of each model was performed within the framework of a systematic grid search method and 5-fold cross-validation in order to obtain the best generalization performance in our high-dimensional dataset. For example, for the KNN classifier, the k value was tested from 1 to 20 with increments of 1; for each k value, accuracy, Kappa coefficient and cross entropy were calculated and the 6 values ​​giving the highest performance were determined, and the Manhattan measure was used as the distance measure. In the Random Forest model, the number of trees (ntree) was tested between 1000 and 10000. As a result of the analysis, using 10000 trees and determining the number of variables considered at each node (mtry) as 10 and the sample size as 1000 optimized the general generalization ability of the model in the best way. For the Naive Bayes classifier, in order to optimize the model without disrupting its basic assumptions, the Laplace correction factor was evaluated from 0.0 to 1.0 with 0.1 increments and the best result was reported when the Laplace factor was 0.3. In the case of using the Support Vector Machine, the regularization parameter C was optimized in the logarithmic range from 0.01 to 100, and the gamma parameter was optimized between 0.0001 and 1.0; the best performance was obtained with C = 10 and gamma = 0.01. These hyperparameter adjustments were meticulously performed in a wide parameter space for each model, and the best performing hyperparameter sets were selected according to the 5-fold cross-validation results. In this way, not only the overall accuracy of the models was ensured, but also the prevention of over-learning and increased generalization ability. All these hyperparameter optimization processes allowed us to evaluate the performance of each model based on objective measures such as Accuracy, Kappa and Cross-Entropy, and were supported by detailed performance tables in the conclusion section of our study. With the parameter settings explained in the model validation, it is expected that the result metrics in feature selection will be improved, and higher estimation results will be obtained. The general metrics used for model validation were Accuracy, Kappa and Cross-Entropy values. In addition, the Sensitivity, Specificity and Accuracy values ​​were calculated to determine which class labels the classification algorithms were more successful in. This process was conducted by creating a sample of equal numbers from the remaining five categories for each special needs category. The newly created sample was created using random subsampling in proportion to the sample sizes of the remaining five categories. In other words, a simple class reduction was performed to calculate the Sensitivity and Specificity values in the form of being in the relevant label class.

## Results

### Results on feature selection

In this study, RFE process was implemented by integrating it with k-fold cross-validation to prevent the accidental elimination of weakly correlated but highly predictive features [112]. In each iteration, the Accuracy, Kappa and Cross-Entropy metrics of the model were monitored; if the removal of a feature caused a decrease of more than 1% in these metrics, the feature in question was added back. For example, Item 7 “Ability to discriminate colors” was retained because it had a low correlation on its own, but when removed, it caused a 4.1% loss of accuracy in the classification of mental retardation. Similarly, Item 23 “Ability to perform multiplication without using hands” was retained because it showed a low correlation but caused a 3.3% loss of accuracy in the classification of specific learning disabilities. Finally, the eliminated features were added back to the model one by one, and it was seen that none of them improved by Kappa > 0.01. This multiple strategy reduced the overall accuracy of the model to 48 critical items out of 171 items, while maintaining the overall accuracy of the model above 90%.

**Fig. 1:**
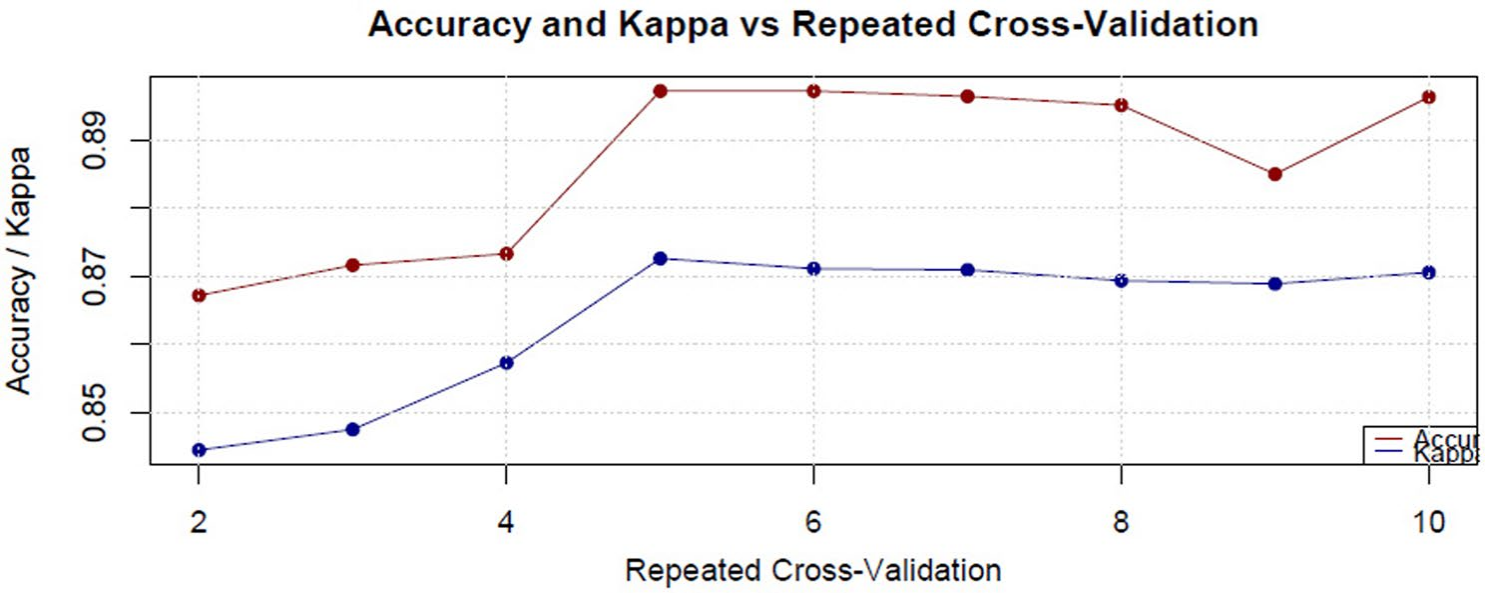
The accuracy and Kappa metrics measure the model accuracy over recursive feature elimination cross-validation iterations

**Fig. 2:**
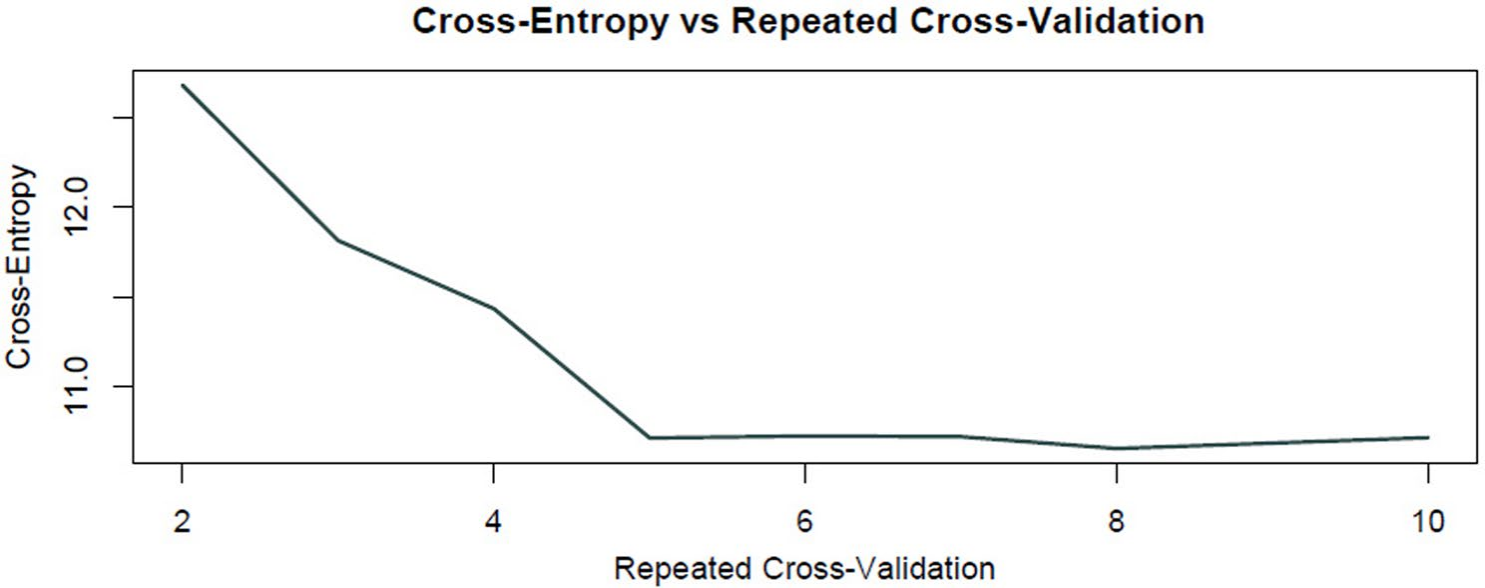
Cross-entropy values ​​that measure model accuracy of recursive feature elimination over cross-validation iterations

Figure 1 shows the effect of the number of variables included in the model on the Accuracy and Kappa metrics depending on the number of k-fold cross-validation iterations in the recursive feature elimination process. Figure 2 reveals the changes in the Cross-Entropy values ​​of the model during the same cross-validation iterations.

In the analyses, the most informative 48 features were selected from the 171 items initially with the RFE method. Model training was performed with this feature subset and the generalizability of the selected 48 features was evaluated with K-fold cross-validation. According to the 5-fold cross-validation results presented in Fig. 1, the Accuracy and Kappa values ​​of the model were obtained as 0.90 and 0.87, respectively; this shows that the model exhibits a high level of consistency in classification performance. Cross Entropy Loss analysis (Fig. 2) was used to measure the agreement between the model’s predictions and the real class labels. In this analysis, no significant decrease was observed in the cross entropy loss during the 5-fold cross-validation process; this reveals whether the model had additional learnable options during the training phase, the decision boundaries were optimized and the risk of overfitting was under control. The feature selection and model validation processes were meticulously separated. First, the most effective 48 features that would increase the classification success of the model were determined with the RFE method; This subset provided high performance values ​​such as AUC = 0.913 and Accuracy = 92.7%. Alternatively, increasing the number of features to 60 caused the accuracy to drop to 85.4%, and decreasing it to 30 caused it to drop to 82.3%. In addition, no significant change was observed in the cross-entropy loss, indicating that the model could not learn additional useful patterns with the current feature set and its performance became stable. After the feature selection process, the model was tested using cross-validation. According to the 5-fold cross-validation results, the average accuracy rate of the model was calculated as 91.8%, its standard deviation as 23.27% and its variance as 0.054. These statistical values ​​show that the model performs similarly on different datasets and does not have high variance. In addition, 5-fold cross-validation was applied to verify that the model did not over-learn and hyperparameter optimization was performed carefully. The minimal difference between the training accuracy (98.2%) and the test accuracy (91.3%) proves that the model does not show a tendency to overfit. Elimination of unnecessary variables with the RFE method has reduced the model complexity and increased its generalization ability. As a result, the low difference between the class labels predicted by the model and the real class labels shows that the classes in the data set can be distinguished with high accuracy. This finding indicates that the model can make reliable and generalizable predictions in preliminary psychological assessment processesThe information regarding this dataset is provided in Table 2.

**Table 2:** Input variables included in the prediction model

Abbreviation	Description
Item 1	Attention to visual stimuli cognitive skill level
Item 2	Classification and matching of objects and pictures skill level
Item 3	Cognitive skill level of ordering events in order of occurrence
Item 4	Imitation of psychomotor skills
Item 5	Cognitive skill level of imitating sounds
Item 6	Skill of playing games with rules
Item 7	Skill of distinguishing colors
Item 8	Visual writing skill
Item 9	Skill level of reading and writing sentences
Item 10	Skill of understanding and summarizing what is read
Item 11	Running and psychomotor balance level
Item 12	Psychomotor coordination level in vertical movements
Item 13	Level of physical balance on two legs
Item 14	Level of physical balance on one leg
Item 15	Ability to provide psychomotor balance in simple games
Item 16	Ability to position objects in a balanced manner
Item 17	Ability to classify entities according to their opposites
Item 18	Ability to count forward rhythmically
Item 19	Ability to recognize and write numbers
Item 20	Ability to distinguish between odd and even natural numbers
Item 21	Ability to distinguish between numbers according to whether they are small or large
Item 22	Ability to perform addition operations with carry on natural numbers
Item 23	Ability to perform multiplication without a hand
Item 24	Ability to perform division without a remainder
Item 25	Ability to perform division with a remainder
Item 26	Ability to solve problems with addition
Item 27	Ability to solve problems with subtraction
Item 28	Ability to solve problems with division
Item 29	Ability to read a clock correctly
Item 30	Ability to recognize geometric shapes
Item 31	Ability to calculate the perimeter of geometric shapes
Item 32	Ability to recognize and use simple fractions
Item 33	Ability to write fractions and perform basic operations with fractions
Item 34	Level of toilet control
Item 35	Level of having basic self-care skills
Item 36	Level of fine motor skills
Item 37	Level of gross motor skills
Item 38	Frequency of using self-care materials
Item 39	Frequency of participating in group activities
Item 40	Ability to choose among options
Item 41	Frequency of experiencing learning difficulties
Item 42	Ability to transfer learned knowledge and skills
Item 43	Ability to follow instructions
Item 44	Ability to use school tools and equipment regularly
Item 45	Ability to pay attention to details
Item 46	Mobility level
Item 47	Ability to hold learning materials at an appropriate distance from the eyes
Item 48	Ability to write words and letters correctly

#### Classification results

The general classification results of the supervised classification methods, KNN Classifier, Random Forest Classifier, Naive Bayes Classifier and Support Vector Classifier algorithms are given in Table 3 with Accuracy, Kappa and Cross-Entropy metrics. The Accuracy, Sensitivity and Specificity values ​​of the classification results obtained using the relevant classification methods for each special needs subclass are presented in Table 3.

**Table 3:** Accuracy, kappa and Cross-Entropy values ​​for KNN classifier, random forest classifier, Naïve Bayes classifier and support vector classifier

Classifier	Accuracy	Kappa	Cross-Entropy
KNN Classifier	0.89	0.86	2.71
Random Forest Classifier	**0.92**	**0.89**	**2.41**
Naive Bayes Classifier	0.90	0.87	2.58
Support Vector Classifier	0.87	0.85	2.77

When Table 3 is examined, it is seen that all supervised classification methods provide high Accuracy and Kappa metrics and result in relatively low cross entropy values. K-Nearest Neighbor Classifier provided 0.89 accuracy, 0.86 kappa and 2.71 cross entropy values. Random Forest Classifier provided the highest Accuracy and Kappa values ​​with 0.92 Accuracy and 0.89 Kappa values ​​and the lowest cross entropy value with 2.41 cross entropy value. As can be understood from these three metrics, it performed classification with higher detection accuracy compared to other classification algorithms. Naive Bayes Classifier provided the best values ​​after Random Forest Classifier with 0.90 Accuracy, 0.87 Kappa and 2.58 Cross Entropy values ​​and reached the classification accuracy. Although the support vector classifier produced a very good class solution for the classification problems with 0.87 Accuracy, 0.85 Kappa and 2.77 Cross Entropy values, it gave the lowest detection rates among the algorithms. It was seen that the Cross Entropy value for SVC had the highest loss rate.

Considering that the class imbalance problem may have misleading effects on the accuracy metric, SMOTE was applied as mentioned in the previous section and the representation of minority classes was increased. In addition, a more comprehensive evaluation of the model performance and performance metrics that measure the model’s prediction success for each class in detail were also included in the analysis. For this purpose, in addition to the Accuracy metric, Precision, Recall and F1-score additional metrics were calculated. Each of the Precision, Recall and F1-score metrics demonstrates the model’s ability to minimize false positive predictions, correctly capture classes and maintain overall balance. Similarly, Cross-Entropy is a metric that measures how reliable the model’s probability estimates are. However, since it does not directly show how well it distinguishes between classes, it was supported by the ROC-AUC score. The overall evaluation of the prediction models resulted in an ROC-AUC value of 91.3% for the Random Forest model, with an average precision of 89.8%, an average recall of 88.4% and an average F1 score of 89.1%. Comparable performance trends were observed for the Naïve Bayes, KNN and Support Vector models, although in some cases with slightly lower maximum precision values. Specifically, the Naïve Bayes model achieved an ROC-AUC of approximately 89.5% and an F1-score of approximately 87.4%, the KNN model achieved an ROC-AUC of approximately 88.3% with an F1-score of 86.5% and the Support Vector model achieved an ROC-AUC of approximately 87.1% with an F1-score of 84.3%. In addition, Precision, Recall and F1-score values ​​for individual classes are presented in Table 4.

**Table 4:** Precision, recall, and F1-Score values for individual classes across RFC, NBC, KNNC, and SVC

Class	Metric	Random Forest	Naïve Bayes	KNN	Support Vector
Mental Disability	Precision	0.8427	0.8300	0.8200	0.8000
	Recall	0.9146	0.9000	0.8900	0.8700
	F1 Score	0.8772	0.8650	0.8530	0.8320
Behavior and Adaptation Problems	Precision	0.8765	0.8600	0.8550	0.8400
	Recall	0.8658	0.8500	0.8400	0.8200
	F1 Score	0.8712	0.8550	0.8470	0.8300
Autism Spectrum Disorders	Precision	0.9250	0.9150	0.9100	0.8850
	Recall	0.9024	0.8900	0.8800	0.8600
	F1 Score	0.9136	0.9020	0.8940	0.8720
Physical Inadequacy	Precision	0.9012	0.8900	0.8800	0.8600
	Recall	0.8902	0.8800	0.8700	0.8400
	F1 Score	0.8957	0.8850	0.8750	0.8500
Visual or Hearing Impairment	Precision	0.9167	0.9100	0.9000	0.8800
	Recall	0.8049	0.7900	0.7800	0.7600
	F1 Score	0.8571	0.8460	0.8360	0.8100
Specific Learning Disability	Precision	0.8652	0.8550	0.8500	0.8300
	Recall	0.9390	0.9250	0.9200	0.9000
	F1 Score	0.9006	0.8890	0.8840	0.8630

Table 4 and the subsequent subsections reveal that the performance metrics demonstrate high overall class discrimination capabilities across all four classification models. The RFC model, with Precision values ranging between 84.2% and 92.5%, is particularly notable for its effectiveness in minimizing false positive predictions. Although the NBC, KNNC, and SVC models exhibit similar trends in Precision, their maximum values are relatively lower. When considering the Recall metrics, all models consistently perform within the 76.0–91.4% range, indicating a stable capacity to correctly identify true positive instances. Furthermore, the F1-scores—which reflect the balance between Precision and Recall—highlight that the RFC model achieves an average performance of 89.1%, while the other models also yield comparably balanced outcomes. These results suggest that the models systematically optimize the control of both Type I and Type II errors. Additionally, the previously calculated ROC-AUC scores for all classifiers further confirm their high discriminatory power. Despite minor variations in specific metric values, each model maintains robust class separation, thereby reinforcing the overall reliability of the predictive framework [113–116].

**Fig. 3:**
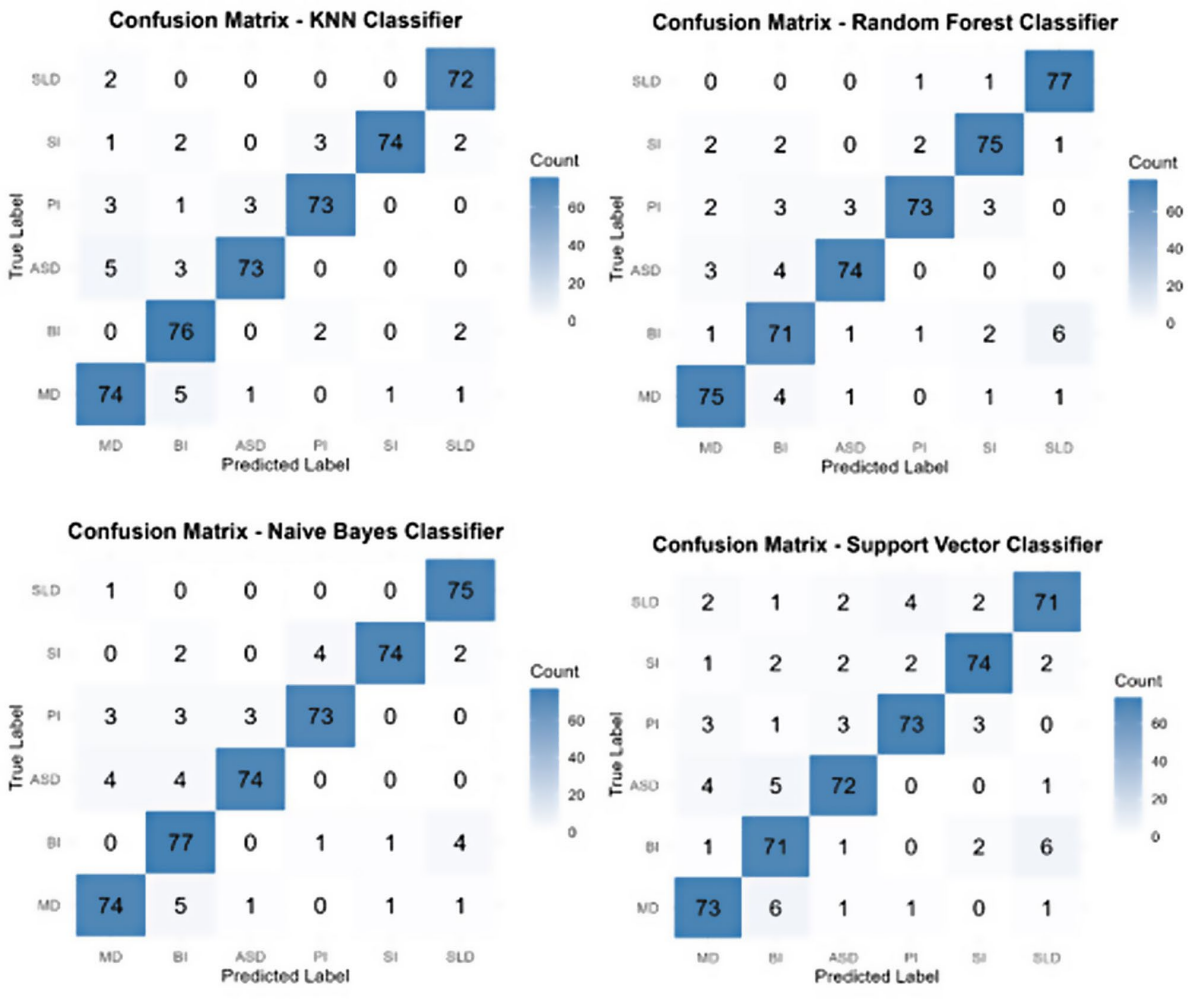
Test dataset complexity matrix results for classification accuracy in KNNC, RFC, NBC and SVC methods (Abbreviations in the matrices are used as MD (Mental Disability), BI (Behavioral Issues), ASD (Autism Spectrum Disorder), PI (Physical Impairment), SI (Sensory Impairment) and SLD (Specific Learning Disability))

Then, in addition to the Kappa metric, the complexity matrix was examined to analyze which classes the model predicted more successfully. The confusion matrices created for the four different classifiers used in this study (KNN, Random Forest, Naive Bayes and Support Vector Classifier) ​​show which diagnostic classes are confused with each other and which types of errors (false positive, (FP) and false negative, (FN)) occur as a result. Having equal sample sizes for all classes in the data set eliminates the effect of shifts caused by class imbalance when interpreting the error rates of the model. In this case, FP and FN rates directly depend on the model’s discrimination between classes, its success in determining decision boundaries and the overlap of features. For example, some models may produce higher FP or FN errors in certain classes due to more uncertainty among classes with similar features. The error analysis over the confusion matrix of each model is detailed in Fig. 3 below.

KNN made some small misses especially in some classes such as ASD and PI. For example, approximately 9 out of 82 real ASD cases were assigned to different classes, and the FN rate was observed around 11%. Despite equal sample size, if the characteristic features of some classes (e.g. ASD or PI) overlap with other classes, the model may miss real examples belonging to these classes. Therefore, for example, a certain proportion of cases in the ASD class may be recorded as FN. By the same logic, KNN may assign cases that do not belong to a class as false positives due to some inter-class similarities. A few errors are observed in common classes such as BI, where examples from other classes are labeled as BI. For example, 5 real ASD and 5 real MD are labeled as BI. As a result, an increase in false positives is observed in the BI class. Similarly, although MD is also recognized at a fairly high rate (74 correct predictions), a small number of cases from some other classes (ASD or PI) could be labeled as MD.

Some rare cases stand out in RF, especially for PI. Of the 82 individuals with PI, 73 were correctly identified, 9 were incorrectly classified, and a FN rate of approximately 10% was observed for PI. This may be due to the overlapping symptoms of PI with BI (3 cases) and ASD (3 cases). However, a decrease in the number of FNs was observed in general compared to KNN. In terms of false positives, errors such as labeling 1 MD and 2 SI cases as well as 6 SLD cases as BI were detected in the BI class, and again, a small number of BI cases (4) were incorrectly labeled as MD in the MD class. RF generally provides more stable results thanks to the consensus of multiple decision trees. Even in the case of equal samples, since there may be variation between trees for some classes (e.g. SI or PI), certain rates of FN and FP may be observed. This may be due to the variation between trees of RF and its limited flexibility, especially in classes with high clinical overlap (BI, ASD, PI). The results seem to be more successful than KNN in balancing both FNs and FPs.

Naive Bayes showed high accuracy by correctly classifying 74 out of 82 cases in the MD class (8 FN); similarly, 74 out of 82 cases were predicted correctly in the ASD class and 8 FNs were produced. This result shows that the model is effective in capturing symptom patterns under the conditional independence assumption, but it also shows the risk of missing real cases, especially in cases where the probabilities between classes are not obvious (e.g., feature similarities between MD and BI). Despite equal sample sizes, insufficient separation of conditional probabilities in classes with similar characteristics led to a FN rate of approximately 10% in both classes. Although Naive Bayes performed well in terms of False Positives by correctly labeling 77 cases in the BI class, 5 BI cases were incorrectly assigned to MD and 4 SLD cases were incorrectly assigned to BI in the class predictions, indicating that the model may experience confusion between classes showing similar symptom patterns (BI ↔ MD/SLD). These FP errors can be thought to be due to the model ignoring hidden dependencies between features, especially in cases where the conditional independence principle does not fully overlap with the real data distribution. For example, clinical/conceptual overlaps between BI and MD or SLD can blur the boundaries in probability-based classification and lead to incorrect assignments. As a result, despite the overall accuracy of the model, the risk of FP in transitions between certain classes stands out as a limitation that should be taken into account, especially in multidisciplinary cases. Although SVC showed a balanced performance in general, significant errors were observed especially in classes with overlapping symptoms such as BI and ASD. In the ASD class, 72 out of 82 real cases were correctly classified, while 10 cases (12.2%) were incorrectly labeled as BI (5), MD (4) and SLD (1). This situation reveals that the model has difficulty distinguishing specific symptoms of ASD such as social communication difficulties from the behavioral features of BI and MD. In the SI class, 74 out of 82 cases were correctly predicted, but 6 cases (7.8%) shifted to PI (2), SLD (2) and other classes. These shifts may be due to SVC’s inability to flexibly handle ambiguous examples due to its rigid decision boundaries. In terms of False Positive (FP) Errors, the BI class is notable: 11 non-BI cases were incorrectly assigned to this class. 6 of them were SLD, 1 was MD, 2 were SI and 2 were PI. This may be related to the broad symptom spectrum of BI overlapping with other classes.

These analyses evaluate model performance not only in terms of overall accuracy but also in terms of the distribution of error types. Strategies that optimize FP and FN rates are critical to reduce the risk of misclassification. Random Forest provides more balanced, reliable and consistent results compared to other models thanks to its ensemble approach, and is particularly superior in reducing FP and FN errors. In summary, under equal sample sizes, the error trends of each model arise only depending on the algorithm’s ability to discriminate between classes and its success in creating decision boundaries. Therefore, since errors resulting from data imbalance cannot be taken into account, FP and FN rates should be evaluated according to the internal performance of the model and the distinguishing features of the relevant classes. In model selection and parameter settings, applying strategies that will minimize these types of errors will play a critical role in reducing the risk of misclassification by strengthening the distinction between classes.

**Fig. 4:**
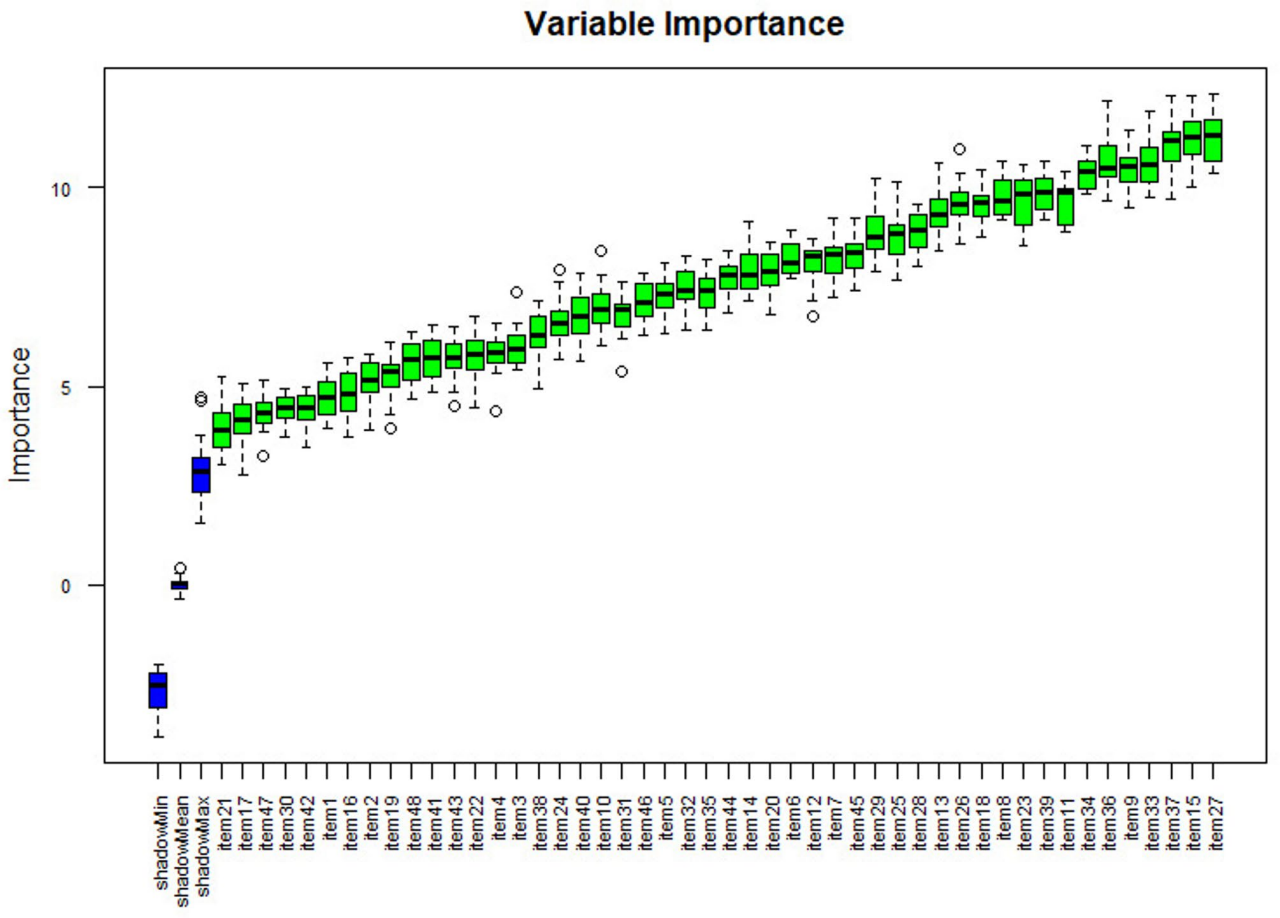
Importance levels of variables for the mental disability diagnostic class

**Fig. 5:**
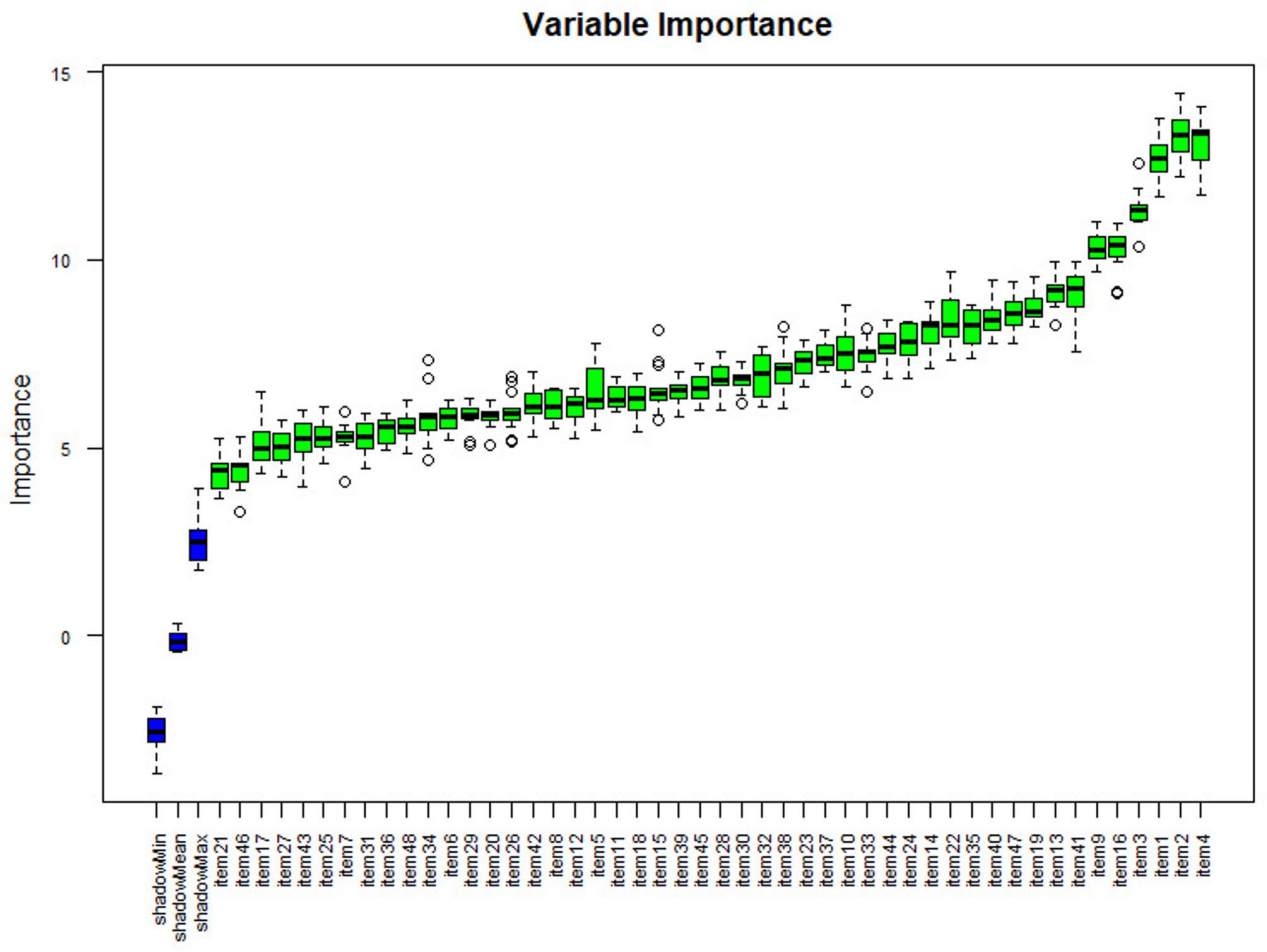
Importance levels of variables for the behavior and adaptation problems diagnostic class

**Fig. 6:**
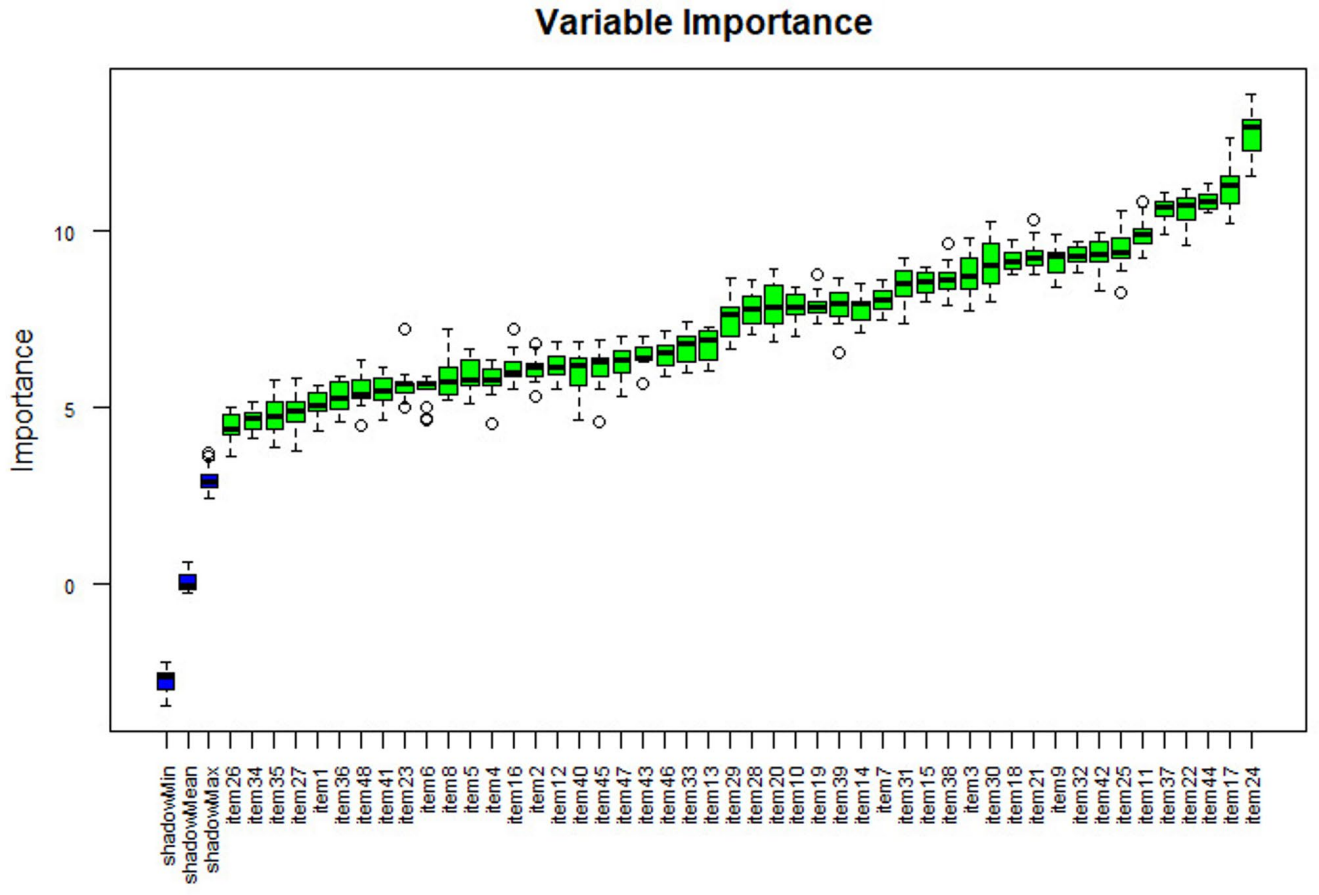
Importance ratings of variables for the autism spectrum disorders diagnostic class

**Fig. 7:**
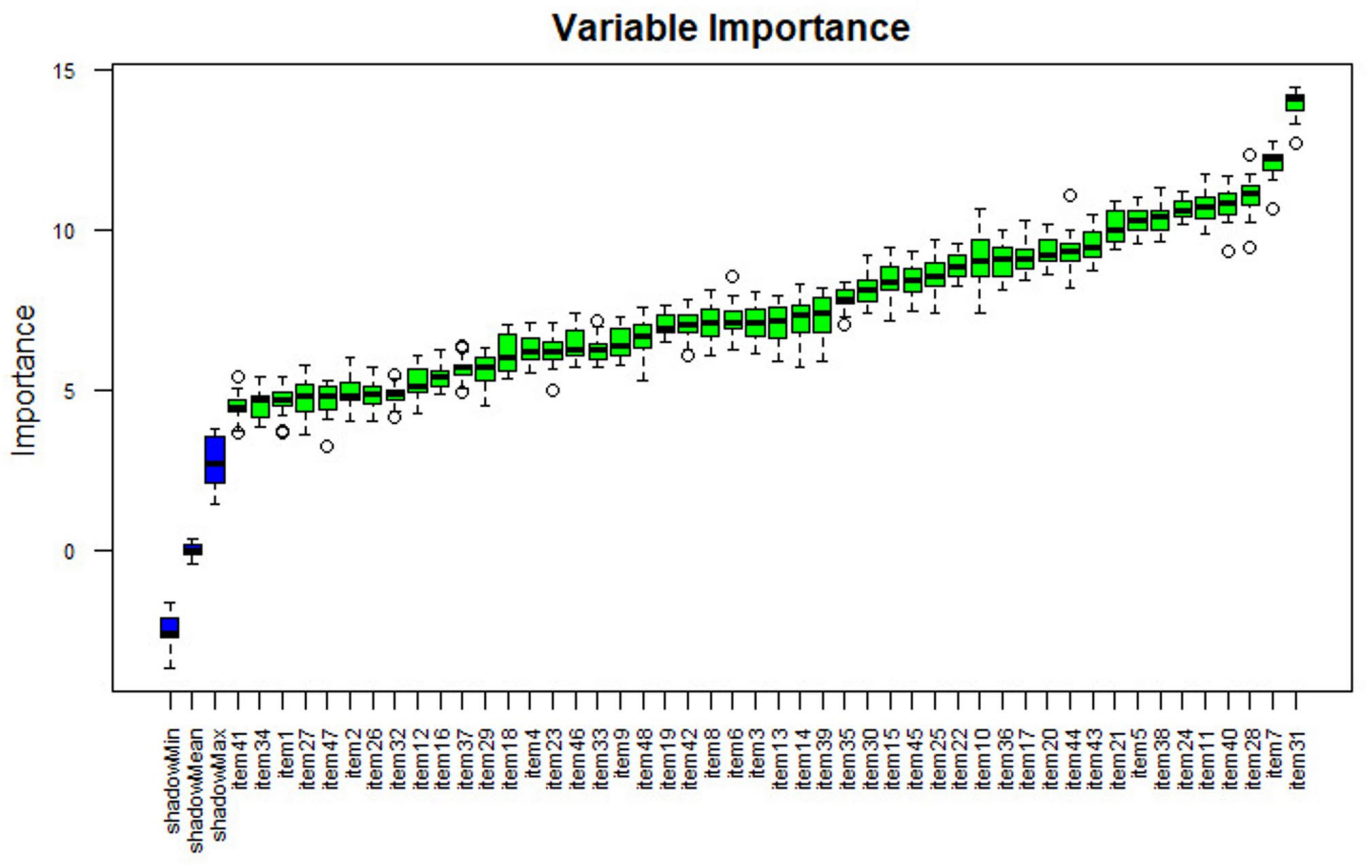
Importance levels of variables for the physical disability diagnosis class

**Fig. 8:**
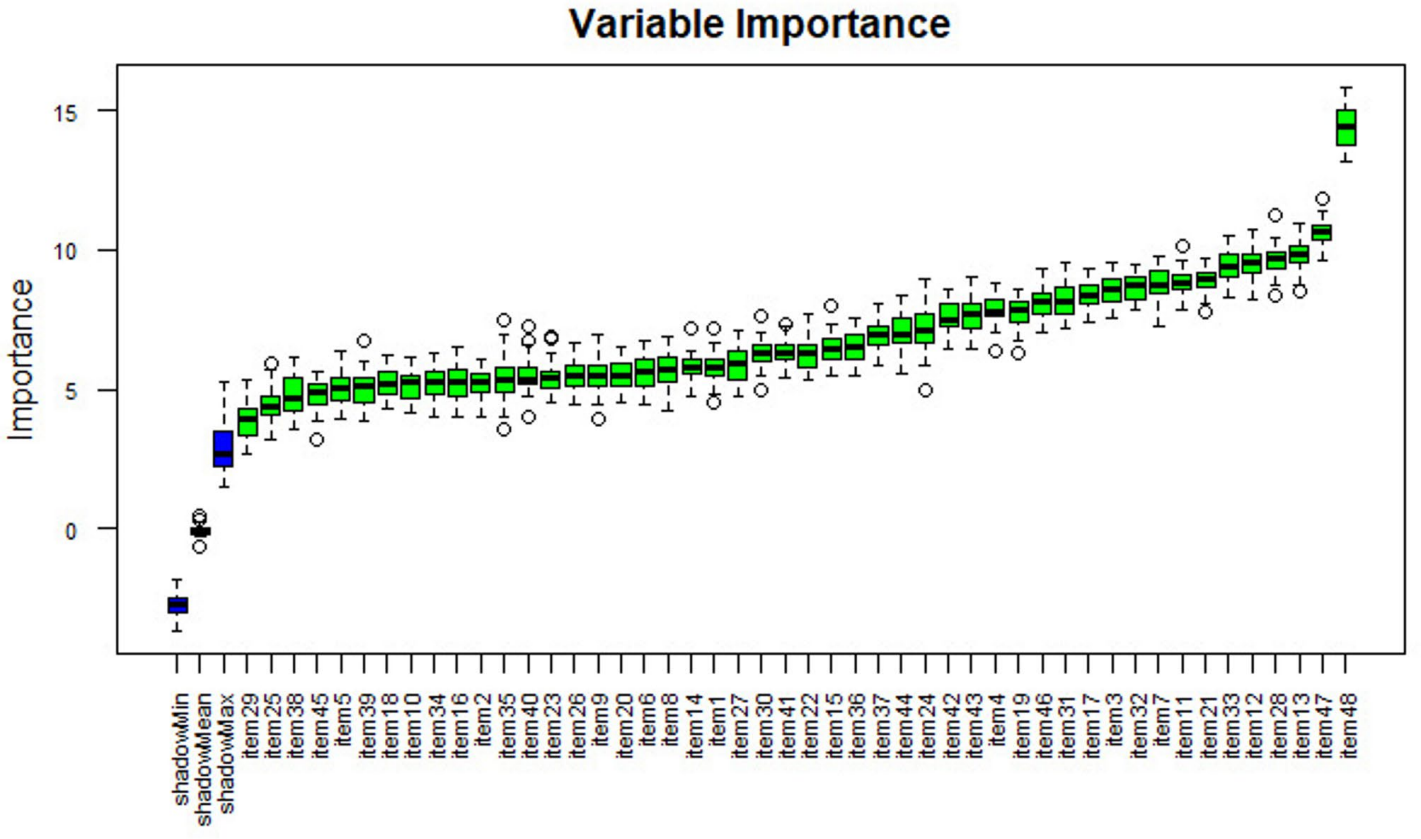
Importance levels of variables for the visual and hearing impairment diagnostic class

**Fig. 9:**
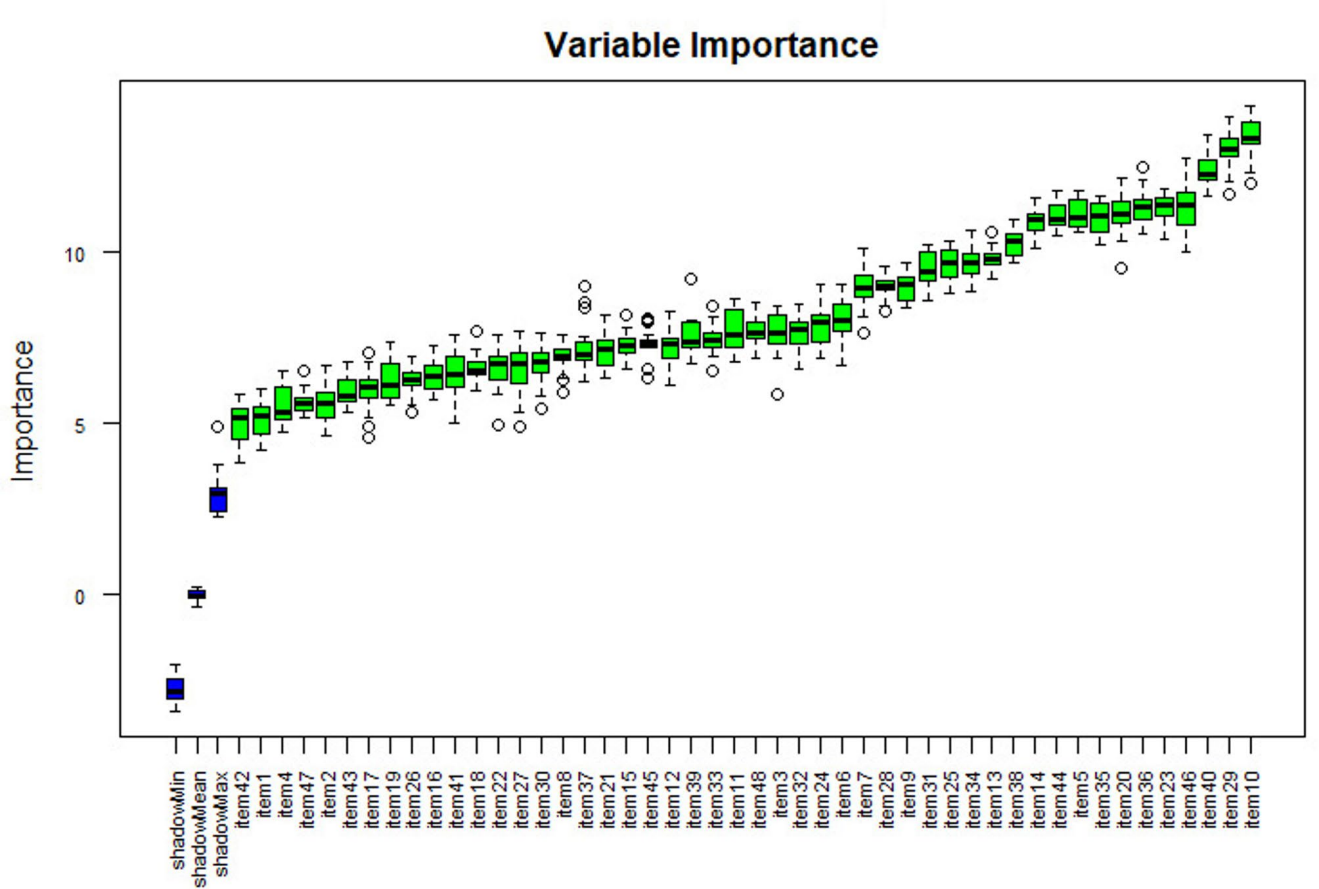
Importance levels of variables for the diagnostic class of specific learning disability

Separate variable importance coefficients were estimated for each diagnosis using the variables determined using the ‘Boruta’ package, which is an R package compatible with the Random Forests algorithm with the highest accuracy values [102]. The results are shown in Figs. 4, 5, 6, 7 and 8, and 9.

As shown in Fig. 4, item 27 has the highest importance coefficient among the items used for the diagnosis of Mental Disability. The importance of all 48 items remained above the shadow values.

As shown in Fig. 5, item 4 has the highest importance coefficient among the items used for the diagnosis of the Behavior and Adaptation Problems diagnostic class. The importance of all 48 items remained above the shadow values.

As shown in Fig. 6, item 24 has the highest importance coefficient among the items used for the diagnosis of the Autism Spectrum Disorders diagnostic class. The importance of all 48 items remained above the shadow values.

As shown in Fig. 7, item 31 has the highest importance coefficient among the items used for the diagnosis of the Physical Disability class. The importance of all 48 items remained above the shadow values.

As shown in Fig. 8, item 48 has the highest importance coefficient among the items used for the diagnosis of the Visual and Hearing Impairment diagnostic class. The importance of all 48 items remained above the shadow values.

As shown in Fig. 9, item 10 has the highest importance coefficient among the items used for the diagnosis of the Specific Learning Disability class. The importance of all 48 items remained above the shadow values.

The importance levels of the variables used to determine each diagnosis based on the importance levels obtained from the analysis and the estimation coefficients obtained based on the importance levels are presented in Table 5. The six models listed in Table 6 were created using the coefficients obtained.

**Table 5:** Importance and prediction coefficients of variables belonging to special needs diagnosis classes

Variables	Mental Disability		Behavior and Adaptation Problems		Autism Spectrum Disorders		Physical Inadequacy		Visual or Hearing Impairment		Specific Learning Disability	
	Significance Coefficient	Coefficient estimates	Significance Coefficient	Coefficient estimates	Significance Coefficient	Coefficient estimates	Significance Coefficient	Coefficient estimates	Significance Coefficient	Coefficient estimates	Significance Coefficient	Coefficient estimates
Item 1	4.68	-0.29	12.91	1.96	5.25	-0.58	4.74	-0.73	5.84	-0.07	5.07	-0.68
Item 2	4.87	-0.22	13.03	3.37	6.18	-0.22	5.0	-0.55	5.4	-0.25	5.73	-0.58
Item 3	5.95	-0.11	11.19	1.94	8.74	0.07	7.13	-0.09	8.58	0.23	8.0	-0.03
Item 4	5.84	-0.11	13.09	6.11	6.06	-0.25	6.23	-0.26	7.88	0.09	5.68	-0.63
Item 5	7.34	-0.05	6.25	-0.06	5.77	-0.26	10.05	0.29	5.03	-0.37	10.99	0.42
Item 6	8.09	0.06	5.55	-0.16	5.63	-0.37	7.05	-0.12	5.65	-0.15	8.29	0.06
Item 7	8.14	0.08	5.09	-0.27	8.15	0.03	12.11	0.75	8.82	0.3	8.77	0.12
Item 8	9.49	0.24	6.0	-0.08	5.67	-0.27	7.05	-0.13	5.73	-0.12	6.9	-0.36
Item 9	10.4	0.39	10.07	1.57	9.13	0.17	6.55	-0.16	5.45	-0.16	9.14	0.24
Item 10	6.77	-0.08	7.58	0.12	7.71	-0.01	8.82	0.1	5.23	-0.3	13.48	1.05
Item 11	9.86	0.26	6.29	-0.06	10.19	0.42	10.48	0.43	8.84	0.33	7.58	-0.09
Item 12	8.13	0.07	6.21	-0.08	6.2	-0.19	5.1	-0.45	9.36	0.47	7.43	-0.12
Item 13	9.33	0.21	8.77	0.7	6.95	-0.02	7.33	-0.08	9.95	0.52	9.87	0.32
Item 14	7.85	0.01	8.12	0.29	8.04	0.03	7.34	-0.08	5.79	-0.08	10.69	0.41
Item 15	11.18	0.83	6.44	-0.03	8.38	0.05	8.33	0.04	6.42	0.03	7.34	-0.17
Item 16	4.76	-0.26	10.27	1.84	6.12	-0.24	5.38	-0.44	5.27	-0.26	6.46	-0.45
Item 17	3.8	-0.49	4.21	-0.47	11.03	0.71	9.25	0.12	8.22	0.21	5.95	-0.56
Item 18	9.44	0.24	6.38	-0.06	9.02	0.16	6.14	-0.34	5.17	-0.3	6.56	-0.42
Item 19	5.24	-0.21	8.72	0.58	7.79	-0.01	6.69	-0.15	7.89	0.15	6.2	-0.54
Item 20	8.06	0.01	5.81	-0.15	7.68	-0.01	9.33	0.13	5.59	-0.15	11.23	0.53
Item 21	6.55	-0.1	0.7	-2.28	9.06	0.16	10.01	0.23	9.1	0.38	7.18	-0.25
Item 22	5.8	-0.14	8.23	0.34	10.8	0.54	8.7	0.09	6.33	0.01	6.77	-0.39
Item 23	9.5	0.24	7.39	0.07	5.62	-0.4	6.24	-0.26	5.44	-0.17	11.49	0.69
Item 24	3.78	-0.58	7.91	0.22	12.8	0.82	10.42	0.38	7.13	0.05	8.23	0.05
Item 25	8.81	0.12	5.0	-0.39	9.46	0.38	8.52	0.09	4.44	-0.48	9.45	0.27
Item 26	9.43	0.21	5.87	-0.09	4.68	-0.72	5.01	-0.49	5.45	-0.17	6.31	-0.51
Item 27	11.32	0.9	4.75	-0.41	5.03	-0.58	4.82	-0.67	5.98	-0.03	6.83	-0.37
Item 28	8.9	0.14	6.59	0.0	7.67	-0.02	11.27	0.6	9.74	0.5	8.9	0.22
Item 29	8.67	0.11	5.77	-0.15	7.54	-0.02	5.91	-0.38	3.71	-1.11	12.97	0.89
Item 30	4.39	-0.32	6.84	0.01	8.99	0.08	8.21	0.02	6.1	0.0	6.88	-0.36
Item 31	6.94	-0.06	5.11	-0.24	8.3	0.04	13.94	1.36	8.14	0.2	9.38	0.27
Item 32	7.34	-0.04	6.98	0.01	9.24	0.17	5.1	-0.47	8.81	0.27	7.8	0.03
Item 33	10.44	0.53	7.84	0.17	6.94	-0.03	6.45	-0.19	9.3	0.45	7.52	-0.1
Item 34	10.37	0.27	5.31	-0.17	4.9	-0.7	4.51	-0.85	5.26	-0.29	9.55	0.32
Item 35	7.53	0.0	8.36	0.41	4.9	-0.65	7.37	-0.01	5.41	-0.24	11.09	0.43
Item 36	10.4	0.27	5.18	-0.21	5.49	-0.57	9.05	0.1	6.56	0.03	11.26	0.68
Item 37	11.16	0.56	7.41	0.08	10.74	0.51	5.43	-0.43	6.91	0.03	6.94	-0.31
Item 38	6.45	-0.1	7.11	0.07	8.68	0.06	10.3	0.3	4.71	-0.46	10.21	0.41
Item 39	9.77	0.25	6.45	-0.01	8.03	0.02	7.37	-0.02	5.12	-0.31	7.51	-0.12
Item 40	6.73	-0.08	8.5	0.42	6.25	-0.13	10.9	0.46	5.42	-0.22	12.19	0.88
Item 41	5.51	-0.17	9.01	0.74	5.61	-0.41	4.47	-1.0	6.31	0.01	6.5	-0.42
Item 42	4.52	-0.3	5.94	-0.08	9.29	0.25	6.98	-0.14	7.72	0.08	5.06	-0.77
Item 43	5.73	-0.15	5.0	-0.4	6.46	-0.07	9.59	0.23	7.74	0.09	5.94	-0.56
Item 44	7.59	0.0	7.84	0.17	10.86	0.56	9.4	0.16	7.03	0.04	10.96	0.42
Item 45	8.39	0.1	6.55	-0.01	6.29	-0.1	8.46	0.05	4.85	-0.39	7.39	-0.13
Item 46	7.16	-0.06	4.03	-0.48	6.61	-0.04	6.32	-0.25	8.12	0.17	11.57	0.76
Item 47	4.29	-0.41	8.5	0.45	6.3	-0.08	4.93	-0.67	10.88	0.55	5.72	-0.63
Item 48	5.28	-0.17	5.19	-0.2	5.5	-0.55	6.61	-0.15	14.4	1.59	7.66	-0.08

**Table 6:** Novel predictive classification models

Special Needs Class Labels	Models
Mental Disability	$\:Model\,1=\,1.464+\:\sum\:_{i=1}^{48}\left[coefficient\:estimation\:of\:item\:i\right]$
Behavior and Adaptation Problems	$\:Model\,2=\,-0.378+\:\sum\:_{i=1}^{48}\left[coefficient\:estimation\:of\:item\:i\right]$
Autism Spectrum Disorders	$\:Model\,3=\,0.674+\:\sum\:_{i=1}^{48}\left[coefficient\:estimation\:of\:item\:i\right]$
Physical Inadequacy	$\:Model\,4=\,0.673+\:\sum\:_{i=1}^{48}\left[coefficient\:estimation\:of\:item\:i\right]$
Visual or Hearing Impairment	$\:Model\,5=\,0.152+\:\sum\:_{i=1}^{48}\left[coefficient\:estimation\:of\:item\:i\right]$
Specific Learning Disability	$\:\:Model\,6=\,0.542+\:\sum\:_{i=1}^{48}\left[coefficient\:estimation\:of\:item\:i\right]$

In order to determine the cut-off values ​​of the models, the cut-off value to be obtained from Model 1 was found to be 0.73 as a result of the multiplication of the fixed coefficient value and the prediction coefficients of each model with the answers given to the item, the cut-off value for Model 1 was found to be 14.70, the cut-off value for Model 2 was found to be 3.52, the cut-off value for Model 4 was found to be 0.49, the cut-off value for Model 5 was found to be 40.61, and the cut-off value for Model 6 was found to be 7.44. The numbers above these cut-off values ​​indicate that the relevant diagnosis can be made, whereas the values ​​below indicate that a sufficient cut-off value for diagnosis cannot be reached. It is estimated that for all diagnoses, those who score above the cutoff value of the relevant diagnosis can be diagnosed based on the answers given by the students who will be newly entered into the system, and those who score below the cutoff value cannot be diagnosed.

## Discussion

The psychological and educational assessment of people with special needs covers various areas. These include cognitive abilities, daily living skills, psychomotor functions and speech and language development. The assessment processes are characterised by their complexity and require a high level of specialist knowledge.

Although standard tests are accepted as the main reference for each special need and developmental area in the assessment process, it is not expected that a single test or a field expert will completely cover all developmental dimensions and diagnostic categories. Moreover, very sensitive and multidimensional diagnostic processes conducted solely based on cross-sectional and instantaneous data, without taking into account the individual’s changes over time, developmental dynamics and adaptation processes, can seriously weaken diagnostic accuracy. Therefore, integrating data obtained from different sources allows for more reliable, comprehensive and accurate results to be obtained by minimizing errors and inconsistencies that may occur in psychological diagnostic procedures. From this perspective, considering assessment processes with a holistic, continuous and dynamic perspective also brings with it the potential to increase the accuracy and reliability of diagnostic processes. The main objective of this study is to develop an innovative approach to overcome the limitations of standardized tests and traditional methodologies used in the educational and psychological assessment of individuals with special needs. Rather than relying only on the obtained test data, the proposed approach foresees systematically integrating information collected from primary data sources such as the individual’s family, educational environment and social environment into the assessment process. Thus, a more comprehensive and contextual perspective is obtained and it is aimed to reach high accuracy rates with the use of machine learning techniques. The proposed predictive assessment model offers a sample procedure through the integration of unstructured data provided by different education systems and levels, thus supporting the standardization, optimization and wide-scale applicability of assessment processes. The application of this model aims to contribute to a better understanding and support of individuals with special needs while providing a comprehensive and guiding framework for educational and psychological assessment practitioners. Our basic assumption is that the proposed approach; standardized tests and traditional assessment methods; It is possible to overcome many problems such as inadequacy in test standardization, error tendencies, limited content, application difficulties, unfavorable evaluation environments, lack of experts, inconsistency between experts, time constraints and limitations in the amount of data obtained, and to produce more effective, comprehensive and predictive results in the educational and psychological evaluation process. In our study, a high-dimensional data set consisting of 171-item psychological evaluation request forms belonging to 1814 people, collected for five years in a psychological evaluation center affiliated with a large educational institution, was used. This data set was meticulously evaluated in order to investigate the effectiveness of the proposed methodology in the classification of six different special needs categories in the psychological diagnosis classification with predictive machine learning approaches. In the first stage, a consistent and appropriate data set was created by ensuring data normalization and class balance in order to obtain more accurate and reliable results; then, different feature combinations were examined in detail and in depth using multiple performance metrics in order to determine the most appropriate features to be used in the training and analysis process of the model. The data set that reached the optimum number of variables with the RFE algorithm was included in the analysis in line with the parameters determined in algorithms such as KNN, Random Forest, Naïve Bayes and Support Vector Machines, and the performance of each model was evaluated comparatively.

The results obtained show that KNN, Random Forest, Naïve Bayes and Support Vector Classifiers exhibit quite strong performances in terms of Accuracy, Kappa and Cross Entropy metrics. Especially, Random Forest Classifier, which reached the highest accuracy, Kappa and lowest cross entropy values, showed superior classification success. These findings are of great importance in terms of practical application; because the observation of high values ​​such as approximately 0.90 in Accuracy and Kappa metrics indicates that the proposed approach offers very low error rates in estimation results and has a high potential to obtain reliable results in clinical applications. In addition, in a study conducted on the evaluation of individuals with special needs, it was reported that approximately 22% of the individuals included in a diagnostic class after psychological and educational evaluations carried out in various centers and health institutions re-applied to the relevant centers due to the problems they experienced after being directed to their social lives and educational processes; 18% of these individuals were included in a different special needs class after being re-evaluated [103]. This situation shows that there may be serious error rates in traditional assessment methods and that the proposed approach can provide much more powerful and predictive results compared to standard tests and classical psychological assessment methods.

Our study, with the predicted classification results obtained, reveals the conceptual importance of psychological and educational evaluations of individuals with special needs, while at the same time, it provides more comprehensive and high-accuracy results beyond the limited number of machine learning-based studies in the literature. Considering that previous studies generally focused on a single special needs class, worked with smaller and limited data sets, and developed models with low detection rates in diagnostic processes [117–119], this study offers a comparative analysis of different models with a more holistic approach. For example [120], classified individuals with ASD and typically developing individuals with 99% accuracy using 93 items in the ADI-R scale [121], presented models that provided over 97% accuracy despite the 72% reduction of ADOS items. In addition, the clinical validity of studies such as the AutMedAI model, which predicts the risk of ASD in children under 2 years of age with 80% accuracy using 28 developmental parameters [122], is controversial, and meta-analyses emphasize that although it shows high performance in controlled data sets, consistency decreases in heterogeneous samples [123]. Examples such as the 75–93% accuracy of deep learning models developed using the ADHD-200 data set in diagnosing ADHD, and the performance of hybrid models supported by expert knowledge reaching up to 93% [124] are similarly supported by the WEL-XGB algorithm, which can detect dyslexia with 98.7% accuracy based on the analysis of reading and writing patterns [125]. It is also reported that artificial intelligence-supported applications such as augmented reality and smart teaching systems contribute to the development of mathematics and reading skills by providing real-time feedback to students [126].

However, although machine learning models reach 95–99% accuracy rates in ASD and dyslexia diagnoses, it is observed that their performance sometimes decreases in real-world data; for example, an ADHD model was reported to provide 90% accuracy on training data, while this rate decreased to 60% on test data [124]. In order to reduce the risk of overfitting and increase the generalizability of the model, it is recommended to use methods such as RFE and cross-validation. At this point, our study aims to integrate multi-class prediction models created with comprehensive and high-dimensional data sets with general assessment and diagnosis processes. The increasing interest in machine learning techniques in the field of educational and psychological assessment reveals their capacity to provide superior accuracy and interpretability compared to traditional methods thanks to the analysis of high-dimensional data [85, 86]. Therefore, in our study, basic algorithms such as KNN, Random Forest, Naïve Bayes and Support Vector Machines were meticulously used to develop new prediction models, both in line with the theoretical foundations in the literature and practical applications [74, 101]. Detailed comparison of these models; evaluation of different parameter settings and feature sets, meticulous planning and implementation of model validation processes have contributed significantly to the overall success of the study. The originality of our study is not only limited to the integration of machine learning techniques, but also the adoption of a holistic and interdisciplinary approach by including unstructured, multidisciplinary preliminary assessment data collected from counseling and guidance centers. Moving beyond the traditional methods based solely on standard test results, the proposed model offers a much broader and more in-depth perspective by integrating the individual’s social environment, developmental history, family structure and expert opinions [25, 66]. This approach allows for more objective, reliable and comprehensive results to be obtained by overcoming the limitations of single measurement tools.

In conclusion, this study presents an innovative and comprehensive methodological framework that goes beyond the boundaries of traditional psychological and educational assessment methods, blends multidisciplinary data with machine learning techniques, and meticulously plans feature selection and model validation processes. Unlike existing approaches, this model, which is not limited to a single type of special needs, narrow-scope environments, or specific developmental periods, aims to increase objectivity and predictive power in clinical diagnosis and educational planning. By critically evaluating the high expertise, cost, and technical infrastructure requirements of artificial intelligence-based tools, a simpler, more economical, and user-friendly alternative has been presented; especially by combining parent, teacher, and counselor views on a single platform, simultaneous analysis of multiple data dimensions has enabled simultaneous evaluation of different special needs classes. It is evaluated that this framework makes significant methodological and applied contributions to the literature, has the potential to form a solid foundation for future research thanks to its resource efficiency and extensibility, and can lead to the determination of new standards in practical applications.

However, the model may erroneously classify an individual who belongs to a real special needs class into a different special needs group, which may lead to the individual being deprived of the specific support they need. For example, incorrectly identifying a student with behavioral problems as a learning disability class may lead to incorrect determination of intervention strategies to be implemented; this may result in ignoring the individual’s unique needs, directing them to inappropriate educational programs, and missing timely intervention opportunities. An even more critical situation occurs in false negative (FN) classification. For example, incorrectly assessing a child with specific developmental disorders such as autism spectrum disorder (ASD) as a general developmental delay may lead to the individual being deprived of vital early intervention and special education services. Such errors may negatively affect the individual’s social, academic, and emotional development, paving the way for greater adaptation and integration problems in later life.

Both types of errors indicate that incorrect classifications among special needs classes prevent individuals from receiving specialized support and interventions. Therefore, in psychological assessment and diagnosis processes, attention should be paid not only to the overall accuracy rate, but also to the classes in which incorrect classification rates are high. In order to avoid misdirection, it is of great importance to consider the unique characteristics of each special needs class in model selection and parameter settings; and to develop correct diagnosis and intervention strategies.

Various strategies can be applied together to reduce misclassification. For example, in order to increase the representativeness of the dataset, collecting more samples for less observed classes such as SI or SLD or applying synthetic data generation techniques such as SMOTE can help reduce the false negative errors of the model in these classes [109]. Similarly, subsampling the majority classes or balancing the classes through class weighting can improve the overall performance [105]. Adjusting the thresholds according to the model output probabilities allows to increase the sensitivity, especially in cases where false negatives are critical, while the increase in false positive rates should also be taken into account [127]. In addition, in approaches where human expert validation is integrated, it becomes possible to re-evaluate false positives and negatives since the models flag the cases that should be examined by experts instead of a single decision maker [128]. Ensemble methods and uncertainty-based strategies can minimize the systematic errors of single models by combining multiple models with consensus or sequential training techniques; In cases where the model is uncertain, delegating the case to expert approval stands out as one of the effective ways to further reduce the risk of misclassification [129].

### Implications and future directions

This study was conducted using a high-dimensional data set with six special needs diagnosis classes. The evaluation of special needs and diagnostic classifications may vary across countries, regions, and institutions. Therefore, future research should consider expanding the scope to include different diagnostic categories and pre-assessment forms, incorporating procedures recommended by both researchers and practitioners. This will help ensure the model’s adaptability and relevance across diverse settings.

Strengthening the technological infrastructure for the integration of machine learning (ML) models into psychological assessment processes is a critical step. In psychological counseling centers, digitizing assessment forms and integrating them into cloud-based systems can provide practitioners with rapid access to data, while making assessment processes more systematic and error-free. In particular, ML-supported digital forms can contribute to achieving more reliable and objective results by enabling instant analysis of data collected from individuals. However, in order for these systems to be successfully implemented, institutions need to conduct infrastructure analyses and determine the technical components needed.

Unstructured forms used in the evaluation process can be further refined into semi-structured or fully structured formats. By iteratively applying these procedures and reducing the number of items, higher detection rates can be achieved. Such a systematic refinement process not only enhances the sensitivity of the screening instruments but also facilitates the development of novel data collection tools that provide clear, step-by-step instructions for assessors. This in turn contributes to standardized, reliable, and user-friendly assessment procedures, thereby improving the overall quality and reproducibility of the collected data.

In terms of real-world application, ML-based systems to be used in schools and psychological counseling centers can automatically analyze form items according to certain criteria and provide specialists with lists of individuals divided into risk groups. In this way, specialists can systematically and data-supportedly determine which students they should focus on first. This allows more effective and timely implementation of intervention plans, especially for counselors working with large student groups. In this way, individuals are provided with the special support they need, while resources are also used in an optimum manner. In order for machine learning models to be successfully implemented, it is of great importance that the systems are not only based on high-performance algorithms, but also have a user-friendly and transparent structure. The interfaces of the proposed ML-based systems should be designed in a way that practitioners can follow the analysis processes step by step and observe in detail the criteria the model bases its decisions on. This approach contributes to the acceleration of diagnosis and support services by providing form optimization for an ML model to be used especially in counseling and research centers. In addition, determining which individuals should be directed to advanced tests as a result of ML analyses makes it possible to use existing human resources more efficiently and optimize early intervention processes.

Future studies can also explore alternative approaches that integrate pre-assessment data from multiple sources, such as behavioral observations, teacher reports, and parental feedback. This integration would enable the acquisition of multifaceted information from diverse psychosocial environments, allowing researchers and practitioners to obtain a more comprehensive understanding of individuals’ needs. By triangulating these various data sources, the effectiveness of psychological and educational assessments can be significantly enhanced, leading to more tailored and effective intervention strategies.

In order for ML-based systems to be used sustainably and effectively in evaluation processes, comprehensive training and professional development programs for practitioners need to be implemented. Regional municipalities and local education authorities can increase the applicability of these systems in local education centers by organizing training programs for psychological counselors on ML-supported forms and data analysis techniques. In this way, practitioners will not only be limited to model outputs, but will also gain competence in correctly interpreting data and making manual corrections when necessary. This will contribute to both technological integration and the continuous professional development of practitioners, thus supporting the success of the system in the long term.

The integration of machine learning with evolving data collection methods holds significant potential for improving the accuracy and efficiency of evaluations across various domains. Future work should not only focus on refining the proposed models and testing their applicability across different educational systems, but also on exploring ways to incorporate real-time feedback loops into the assessment process. By validating the effectiveness of these models in real-world applications, researchers can further contribute to the development of personalized and inclusive assessment practices that are sensitive to the diverse needs of individuals with special needs. This iterative approach may also lead to continuous improvements in the data collection tools and analytical methods used in the field.

Finally, in order for ML systems to be applied in psychological and educational environments, full compliance with ethical and legal regulations is required. For example, ML-based psychological assessment forms to be used in a university hospital or private counseling center should be meticulously examined and approved by ethics committees and integrated into the system. In this process, providing experts with manual intervention mechanisms that can correct the model’s misclassifications will both increase the reliability of the model and contribute to the protection of ethical principles. Thus, incorrect assessments of individuals will be prevented, and both the correct determination of individual needs and the effectiveness of intervention processes will be ensured.

In addition, designing systems in accordance with data security, privacy, and transparency criteria plays a critical role in gaining the trust of all stakeholders. Protecting the rights of both practitioners and individuals being evaluated is a fundamental prerequisite for the successful integration of future systems. In this context, future studies should focus on developing more comprehensive and interactive assessment tools by continuously updating ML models, ensuring compliance with local regulations, and incorporating user feedback into the system development process. In conclusion, the integration of machine learning techniques with emerging data collection methods has great potential to increase the accuracy and efficiency of assessment processes. Future studies should focus on improving the proposed models, testing their applicability in different educational systems, and validating their effectiveness in real-world applications. Such advances will contribute to the development of more personalized and inclusive assessment practices for individuals with special needs.

In addition, during the integration of ML-based systems into psychological and educational assessment processes, strategies should be implemented to minimize the risk of misclassification. The model’s erroneous inclusion of an individual belonging to a real special needs class in a different class may result in the individual being deprived of the specific support they need. For example, erroneously identifying a student with behavioral problems as a learning disability class may lead to incorrect determination of intervention strategies, ignoring the individual’s unique needs, and directing them to inappropriate educational programs. Furthermore, in cases of false negative (FN) classification, a child with specific developmental disorders such as autism spectrum disorder (ASD) may be mistakenly evaluated as a child with general developmental delay, which may result in the individual being deprived of early intervention and special education services. Such errors negatively affect the individual’s social, academic, and emotional development, paving the way for greater adaptation and integration problems in later life.

Various strategies can be applied together to reduce misclassification. For example, in order to increase the representativeness of the dataset, collecting more samples for less observed classes (e.g. SI or SLD) or using synthetic data generation techniques such as SMOTE can be effective in reducing the false negative errors of the model in these classes [109]. Similarly, subsampling the majority classes or balancing the classes by class weighting can improve the overall model performance [105]. Adjusting the threshold according to the model output probabilities can increase the sensitivity, especially in cases where false negatives are critical, while also taking into account the increase in false positive rates [127]. Furthermore, in approaches where human expert validation is integrated, the models flag cases that should be reviewed by experts rather than a single decision maker, allowing false positives and negatives to be re-evaluated [128]. Ensemble methods and uncertainty-based strategies can minimize the systematic errors of individual models by combining multiple models with consensus or sequential training techniques; Especially in cases where the model is uncertain, delegating the case to expert approval stands out as one of the effective ways to further reduce the risk of misclassification [129].

This expanded framework offers important steps towards increasing both accuracy and effectiveness in educational and psychological assessment processes by integrating both technological and application-oriented approaches. Thus, comprehensive and holistic solutions can be developed to increase the quality of life of individuals with special needs by ensuring both the correct identification of individual needs and the effectiveness of intervention strategies.

Future studies should focus on creating data sets that cover a wider range and the adaptability of models to different contexts. In addition, integration models that comply with ethical and technical standards should be developed to ensure that machine learning-based systems are effectively integrated into human-centered decision processes.

The R codes and data preprocessing steps used in the study can be shared as open source on GitHub. This allows other researchers to implement the model in their own data sets in a few steps. In addition, models using such algorithms can be easily added to software used in institutions such as Guidance and Research Centers with API integration.

In addition to the machine learning (ML) methods used in this study, approaches such as Deep Learning and Transformer-based models can also be suggested to improve the classification performance of individuals with special needs. These models have the potential to capture complex relationships in data and model feature interactions more effectively [130]. ​​Variants of the Transformer architecture optimized for tabular data (e.g., TabTransformer) are quite successful in modeling the interactions of categorical and numerical features [131]. These methods can create richer representations by learning semantic embeddings of categorical features. At the same time, in our study, they can capture cross-relationships between features across diagnostic classes with attention mechanisms (e.g., the connections between “language skills” and “social interaction”). The 171 categorical items in the educational assessment request forms we used in our study can be represented by embedding layers, analyzed with multi-head attention mechanisms, and used to predict diagnostic classes.

Another class of methods we can suggest are Multilayer Perceptrons (MLP) and Deep Neural Networks. Simple Multilayer Perceptrons (MLP) can be used to model nonlinear relationships in normalized datasets [132]. They capture abstract relationships in data by performing hierarchical feature extraction with hidden layers. For example, the normalized 48 features in our study can be fed to a network with 3–5 hidden layers to perform classification. Dropout or L2 Regularization can be added to prevent over-learning [133]. Again, instead of the existing Random Forest model that gave the best results in our study, LightGBM or XGBoost can be used to further increase accuracy with hyperparameter optimization (e.g. learning rate, max_depth) [134, 135]. Another suggestion for future practitioners and researchers is to use the Feature Extraction method with Autoencoders. Autoencoders provide feature compression and denoising by representing the data in a low-dimensional latent space. Thus, it creates more robust representations in noisy or incomplete data and the extracted latent features can be used in training classifiers [136]. In our application, 171-item raw data can be reduced to a 20–30 dimensional latent space with an autoencoder and given as input to models such as Random Forest or SVM. All these approach suggestions can significantly increase the impact of the current study by providing higher accuracy, cultural adaptation and ethical transparency in the evaluation of individuals with special needs.

## Limitations

The findings of this study show that high classification accuracy can be achieved using well-optimized models powered by specific parameter settings. The applied methods and rigorous parameter settings reveal that the performance achieved during the training process of the model is applicable not only to laboratory conditions but also to real-world data within certain limits. However, some limitations included in the scope of the study must be acknowledged.

Firstly, the study provides an alternative roadmap to standard tests in the psychological and educational assessment of individuals with special needs; however, the generalizability of the findings may be limited by the dataset and sample characteristics used. This situation is the result of analyses based only on a specific geographical region or sample characteristics. Therefore, it is of great importance that future studies increase the applicability of this approach by verifying the findings among larger populations and different datasets. In addition, factors such as the sample size of the dataset used, the demographic characteristics of the sample, and the sample diversity may directly affect the external generalizability of the model. Therefore, examining larger datasets and various sample characteristics will contribute to further strengthening the scope and reliability of the model.

Secondly, the approach used in the study is based on optimizing the classification accuracy with the k-fold cross-validation method. This method has been implemented meticulously to show that the model is consistent during the training process and that the risk of overfitting is minimized. However, in real-world scenarios, the constant variation in data distributions and conditions may cause the model not to perform as expected under different conditions. For example, significant differences in the accuracy rates obtained by the model may be observed in different regions or in groups with different demographic characteristics. Therefore, testing the proposed approach on a larger scale, longitudinal and different data sets will play a critical role in determining the robustness and adaptability of the model.

Thirdly, the study emphasizes the cost and time efficiency advantages provided in updating the preliminary assessment forms compared to standard tests. Although these advantages are especially important in rapid decision-making and early intervention processes, they are directly related to the quality and reliability of the data sources used. The consistency, validity and up-to-dateness of the information provided by different data sources are important factors that can affect the accuracy of the overall results of the study. Therefore, future research should develop comprehensive methods to ensure data consistency and increase validity by conducting comparative analyses between different data sources. Thus, it will be possible to obtain more reliable results in both theoretical and practical applications of the model. Finally, although the proposed method addresses some practical difficulties associated with standardized tests (e.g. outdated content, high development costs, and implementation difficulties), it does not completely eliminate the need for standardization in assessment processes. The consistency and comparability features offered by standardized tests, although in some cases inadequate, remain important as a fundamental component of assessment processes. In this context, additional research is needed on how they can be integrated or complemented with traditional methods in various educational and psychological settings. Such studies have the potential to both eliminate the shortcomings of existing methods and facilitate the integration of new approaches.

Machine learning-based assessment models offer the opportunity to make faster and more efficient diagnoses by supporting psychological and educational screening processes. However, during the integration of these approaches into real-world applications, it is necessary to consider not only high classification accuracy, but also important factors such as the generalization ability of the model, ethical compliance, and application reliability. Various difficulties that may be encountered in the field of application require meticulous evaluations on how to interpret the outputs of the model and in which cases manual intervention will be needed. In this context, adopting a critical perspective while evaluating the findings of the study and considering their applicability is vital for the success of both academic and practical studies.

The dataset used in the study is limited to a specific geographic region and children between the ages of 6 and 12, which makes it impossible to determine with certainty whether the model will perform similarly on different populations or more heterogeneous groups. It has been reported that in machine learning models trained with similar data, models optimized for a specific group do not exhibit the same level of accuracy on individuals with different socioeconomic or cultural structures [137]. Especially in psychological assessments, the variability of diagnostic criteria in different regions and the different interpretation of the same symptoms are among the factors that can seriously affect the generalizability of the model. For this reason, in order to increase the generalizability of the model, it is essential to conduct multi-center studies among different populations and education systems and systematically test the consistency of the model on various cultural and demographic groups. The critical role of diagnostic assignments made by experts in the dataset on which the model is trained may lead to some limitations in terms of ethics and objectivity. In psychological assessments, the accuracy rates of expert opinions may vary, and it is also possible for the same individual to be placed in different categories by different experts [138]. This situation also brings with it the risk that certain biases may be reflected in the model’s learning process. It has been shown in the literature that factors such as socioeconomic status, ethnicity and gender can affect expert decisions in psychological and educational assessment processes [139]. Therefore, in order to minimize bias in the model’s learning process, it is of great importance that labeling processes are verified by more than one expert and data editing techniques are applied to reduce systematic bias [78].

One of the main problems faced by machine learning models is the risk of overfitting. Overfitting of the model to certain patterns and noises in the dataset on which it was trained causes the model to fail to show the expected performance on new and different datasets [132]. In this study, k-layered cross-validation and iterative feature selection methods were applied to minimize the risk of overfitting. However, it is not possible to determine the real-world performance of the model without testing it on various datasets [76, 77]. Future research will provide a critical step in the transition to real-world applications by testing the model on independent datasets in different centers and analyzing the performance stability in detail. Finally, the ethical and legal compliance of ML-based assessment models is an important element that determines their reliability in psychological and educational applications. In order for the results predicted by a model to be used in decision-making processes, it must first be meticulously evaluated by ethics committees and expert boards [140]. In order to increase the transparency of the model, it is important to provide comprehensive information to users about the data on which the system was trained, the criteria on which it made decisions, and potential error rates. In addition, structural measures should be taken to correct the model’s misclassifications, support it with expert opinion, and, when necessary, activate manual intervention mechanisms [141]. The findings show that it has the potential to support decision-making processes in psychological assessment and educational environments. However, during the integration of these systems into real-world applications, issues such as generalization, ethical compliance, and model reliability should be considered in detail. It is expected that future studies will contribute to increasing the applicability of the model in various clinical, academic, and educational environments by testing it on different data sets. In this context, beyond the high classification accuracy provided by the model, meticulous consideration of potential risks and limitations that may be encountered in practice will further increase the success and reliability of the model. In summary, the fact that the dataset used in the study is limited to a specific geographic region and children between the ages of 6 and 12 creates uncertainties about how the model will perform in different populations, and therefore, the importance of multi-center studies with samples with different demographic characteristics is emphasized. Such studies will not only test the cultural and demographic consistency of the model, but will also allow the development of strategies to reduce biases and prejudices that may be encountered in practice.

The findings of the study show that not only can the model achieve high classification accuracy, but also that innovative approaches to support the psychological and educational assessment processes of individuals with special needs can overcome the inadequacies of current standard methods. However, comprehensive, multicenter and interdisciplinary studies should be conducted in the future to increase the applicability of these findings to larger populations, ensure diversity in data sets, minimize biases of the model and ensure full compliance with ethical standards.

## Conclusions

This study presents a predictive assessment model aimed at standardizing and optimizing evaluation processes by utilizing unstructured primary data, which vary across different education systems and levels. The proposed framework seeks to enhance psychological and educational assessment practices, ultimately improving the understanding and support provided to individuals with special needs. The results indicate that the input dataset achieved high classification accuracy with selected combinations of variables. The analysis revealed favorable outcomes at a relatively low computational cost. Among the classifiers tested—K-Nearest Neighbors, Random Forest, Naive Bayes, and Support Vector Machines—the Random Forest classifier demonstrated the highest accuracy and Kappa scores, alongside the lowest cross-entropy value. These metrics, with accuracy and Kappa values approaching 90%, suggest that the proposed approach can produce highly accurate predictions with low error rates. These findings indicate that the proposed approach has the potential to provide more effective, comprehensive and accurate results compared to traditional assessment methods and standardized tests. It also highlights the potential for machine learning techniques to improve psychological and educational evaluations for individuals with special needs. Furthermore, it addresses key challenges associated with traditional approaches, such as high error rates in initial classifications, which often require reevaluations and reclassification. Despite these promising results, it is important to recognize that traditional assessment methods remain vital. Further research is needed to validate and improve the proposed model on different datasets and in various contexts. The study emphasizes the potential of integrating machine learning with primary data sources to enhance prediction accuracy and reduce inconsistencies typically found in multidisciplinary evaluation processes. By offering objective data collection methods, the proposed approach aims to improve the reliability and validity of assessments. Additionally, the study underscores the importance of multidisciplinary teamwork in enhancing the quality and reliability of the evaluation process. The proposed model aims to facilitate such collaborations by providing data-driven, objective insights to minimize inconsistencies. In conclusion, this study offers a promising new approach to improving the psychological and educational evaluation of individuals with special needs. While the proposed model demonstrates potential for enhancing accuracy and comprehensiveness, further validation and exploration are necessary to confirm its broader applicability. This research contributes to ongoing efforts to refine assessment methodologies and better meet the needs of individuals with special needs.

## Data Availability

The datasets generated and analyzed during the current study are available from the corresponding author on reasonable request.
